# The Mathematics of Four or More N-Localizers for Stereotactic Neurosurgery

**DOI:** 10.7759/cureus.349

**Published:** 2015-10-13

**Authors:** Russell A Brown

**Affiliations:** 1 Software Development, High Technology

**Keywords:** stereotactic neurosurgery, stereotactic radiosurgery, image guidance, image-guided, computed tomography, magnetic resonance imaging, positron emission tomography (pet), n-localizer, medical imaging, brain imaging

## Abstract

The mathematics that were originally developed for the N-localizer apply to three N-localizers that produce three sets of fiducials in a tomographic image. Some applications of the N-localizer use four N-localizers that produce four sets of fiducials; however, the mathematics that apply to three sets of fiducials do not apply to four sets of fiducials. This article presents mathematics that apply to four or more sets of fiducials that all lie within one planar tomographic image. In addition, these mathematics are extended to apply to four or more fiducials that do not all lie within one planar tomographic image, as may be the case with magnetic resonance (MR) imaging where a volume is imaged instead of a series of planar tomographic images. Whether applied to a planar image or a volume image, the mathematics of four or more N-localizers provide a statistical measure of the quality of the image data that may be influenced by factors, such as the nonlinear distortion of MR images.

## Introduction

**Figure 1:**
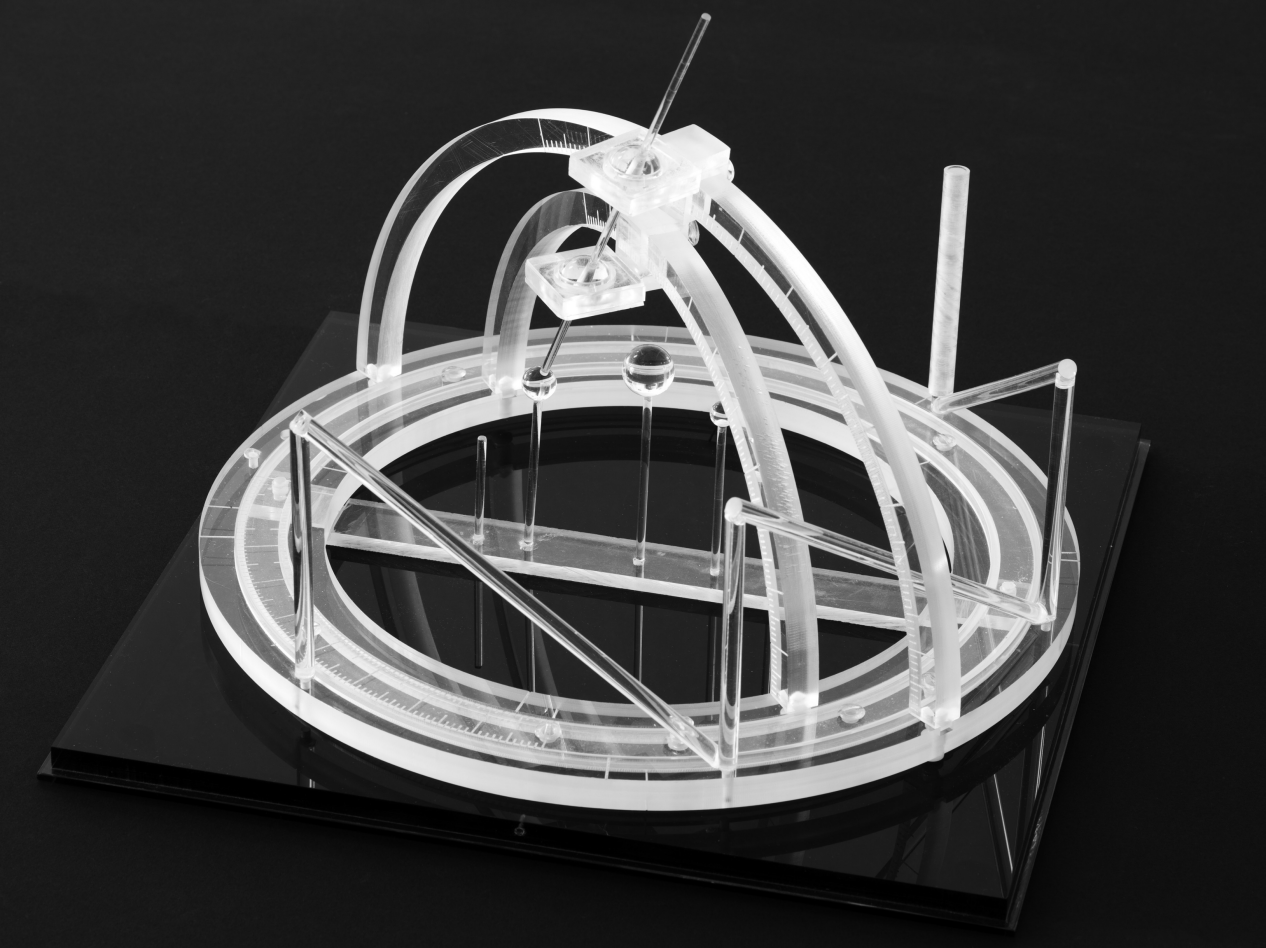
Three N-Localizers Attached to a Stereotactic Frame Three N-localizers are attached to this stereotactic frame and are merged end-to-end such that only seven rods are present. The vertical rod at the right rear of the frame is larger in diameter than the other rods. This large rod facilitates unambiguous interpretation of the fiducial circles and ellipses that the seven rods create in a tomographic image, as explained in the legend to Figure 5.

The N-localizer is a device that may be attached to a stereotactic frame (Figure 1) in order to facilitate image-guided neurosurgery and radiosurgery using tomographic images that are obtained via computed tomography (CT), magnetic resonance (MR) or positron emission tomography (PET) [1]. The mathematics of the N-localizer have been discussed previously [2]. The remainder of this Introduction will review the mathematics of three N-localizers in preparation for the presentation of the mathematics of four or more N-localizers in the Materials and Methods.

**Figure 2:**
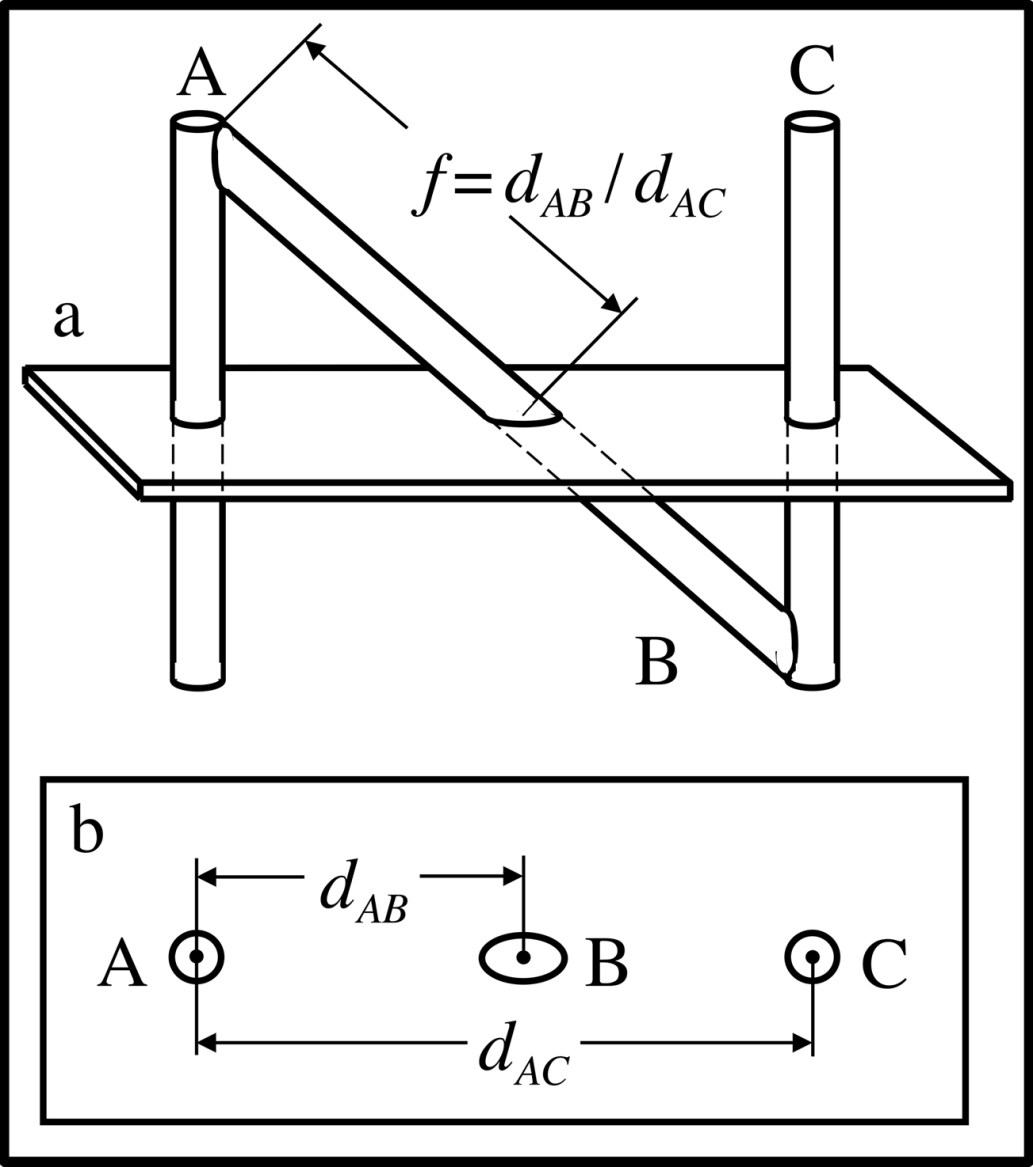
Intersection of the Tomographic Section with the N-Localizer $\mathbf{(a)}$ Side view of the N-localizer. The tomographic section intersects the rods $\mathrm A$, $\mathrm B$, and $\mathrm C$. $\mathbf{(b)}$ Tomographic image. The intersection of the tomographic section with the rods $\mathrm A$, $\mathrm B$, and $\mathrm C$ creates fiducial circles $\mathrm A$ and $\mathrm C$ and fiducial ellipse $\mathrm B$ in the tomographic image. The distance $d_{AB}$ between the centers of circle $\mathrm A$ and ellipse $\mathrm B$ and the distance $d_{AC}$ between the centers of circles $\mathrm A$ and $\mathrm C$ are used to calculate the ratio $f=d_{AB}/d_{AC}$. This ratio represents the fraction of diagonal rod $\mathrm B$ that extends from the top of rod $\mathrm A$ to the point of intersection of rod $\mathrm B$ with the tomographic section.

The N-localizer comprises a diagonal rod that extends from the top of one vertical rod to the bottom of another vertical rod (Figure 2). Assuming for the sake of simplicity that the two vertical rods are perpendicular to the tomographic section, the cross section of each vertical rod creates a fiducial circle and the cross section of the diagonal rod creates a fiducial ellipse in the tomographic image, as shown in Figure 2b. As the tomographic section moves from the top of the N-localizer towards the bottom of the N-localizer*, *i.e. towards its point of attachment to the stereotactic frame (Figure 1), the ellipse $\mathrm B$ will move away from circle $\mathrm A$ and toward circle $\mathrm C$. The relative spacing between these three fiducials permits precise localization of the tomographic section with respect to the N-localizer. The distance $d_{AB}$ between the centers of circle $\mathrm A$ and ellipse $\mathrm B$ and the distance $d_{AC}$ between the centers of circles $\mathrm A$ and $\mathrm C$ are used to calculate the ratio $f=d_{AB}/d_{AC}$. This ratio represents the fraction of diagonal rod $\mathrm B$ that extends from the top of vertical rod $\mathrm A$ to the point of intersection of rod $\mathrm B$ with the tomographic section. These linear geometric relationships exist due to the properties of similar triangles and are valid even if the vertical rods are not perpendicular to the tomographic section [3].

It is convenient to ignore the thickness of the tomographic section and to approximate the tomographic section as an infinitely thin plane. This "central" plane lies midway between the top and bottom halves of the tomographic section, analogous to the way that a slice of cheese is sandwiched between two slices of bread. The central plane approximation is susceptible to error because of the partial volume effect that derives from the several-millimeter thickness of the tomographic section [4-5]. The partial volume effect prevails because any structure that passes partially into the tomographic section but does not span the full thickness of that section may be visible in the tomographic image. Hence, the position of that structure is determined to only a several-millimeter error that is a well-known limitation of tomographic imaging. In the following discussion, the term "tomographic section" will be used as an abbreviation for the term "central plane of the tomographic section."

**Figure 3:**
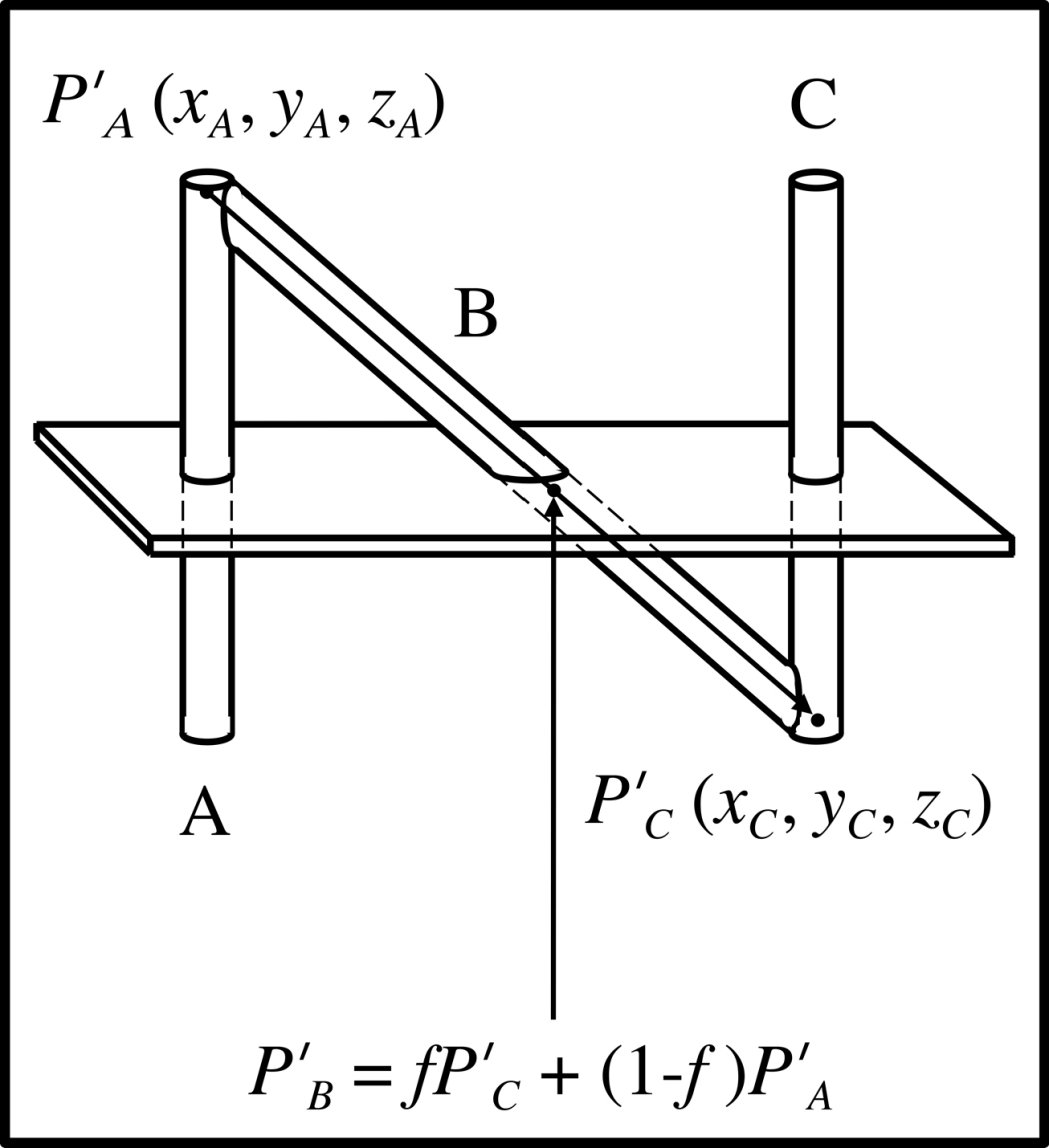
Calculation of the Point of Intersection Between the Rod B and the Tomographic Section The long axis of rod $\mathrm B$ is represented by a vector that extends from point ${P}'_{A}$ at the top of rod $\mathrm A$ to point ${P}'_{C}$ at the bottom of rod $\mathrm C$. The $\left(x_{A},y_{A},z_{A}\right)$ coordinates of point ${P}'_{A}$ and the $\left(x_{C},y_{C},z_{C}\right)$ coordinates of point ${P}'_{C}$ are known from the physical dimensions of the N-localizer. Hence, the ratio $f=d_{AB}/d_{AC}$ may be used to blend the $\left(x_{A},y_{A},z_{A}\right)$ and $\left(x_{C},y_{C},z_{C}\right)$ coordinates of points ${P}'_{A}$ and ${P}'_{C}$ via linear interpolation as indicated by Equations 1 and 2. This interpolation calculates the $\left(x_{B},y_{B},z_{B}\right)$ coordinates of the point of intersection ${P}'_{B}$ between the long axis of rod $\mathrm B$ and the tomographic section.

The fraction $f$ is used to calculate the $\left(x,y,z\right)$ coordinates of the point of intersection ${P}'_{B}$ between the long axis of rod $\mathrm B$ and the tomographic section (Figure 3). In this figure, points ${P}'_{A}$ and ${P}'_{C}$ represent the beginning and end, respectively, of the vector that extends from the top of rod $\mathrm A$ to the bottom of rod $\mathrm C$. This vector coincides with the long axis of rod $\mathrm B$. The $\left(x_{A},y_{A},z_{A}\right)$ coordinates of the beginning point ${P}'_{A}$ and the $\left(x_{C},y_{C},z_{C}\right)$ coordinates of the end point ${P}'_{C}$ are known from the physical dimensions of the N-localizer. Hence, linear interpolation may be used to blend points ${P}'_{A}$ and ${P}'_{C}$ to obtain the $\left(x_{B},y_{B},z_{B}\right)$ coordinates of the point of intersection ${P}'_{B}$ between the long axis of rod $\mathrm B$ and the tomographic section

$${P}'_{B}={P}'_{A}+f\left({P}'_{C}-{P}'_{A}\right)=f{P}'_{C}+\left(1-f\right){P}'_{A}\;\;\;\;\;\;\;\;\;\;\left(1\right)$$

The vector form of Equation 1 shows explicitly the $\left(x,y,z\right)$ coordinates of points ${P}'_{A}$, ${P}'_{B}$ and ${P}'_{C}$

$$\begin{bmatrix}x_{B} \; y_{B} \; z_{B}\end{bmatrix} = f \begin{bmatrix}x_{C} \; y_{C} \; z_{C}\end{bmatrix} +\left(1-f \right) \begin{bmatrix}x_{A} \; y_{A} \; z_{A}\end{bmatrix}\;\;\;\;\;\;\;\;\;\;\left(2\right)$$

Equation 1 or 2 may be used to calculate the $\left(x_{B},y_{B},z_{B}\right)$ coordinates of the point of intersection ${P}'_{B}$ between the long axis of rod $\mathrm B$ and the tomographic section. The point ${P}'_{B}$, which lies on the long axis of rod $\mathrm B$ in the three-dimensional coordinate system of the N-localizer, corresponds to the analogous point $P_{B}$, which lies at the center of ellipse $\mathrm B$ in the two-dimensional coordinate system of the tomographic image (Figure 2b). Hence, there is a one-to-one linear mapping between a point from the N-localizer and a point from the tomographic image.

**Figure 4:**
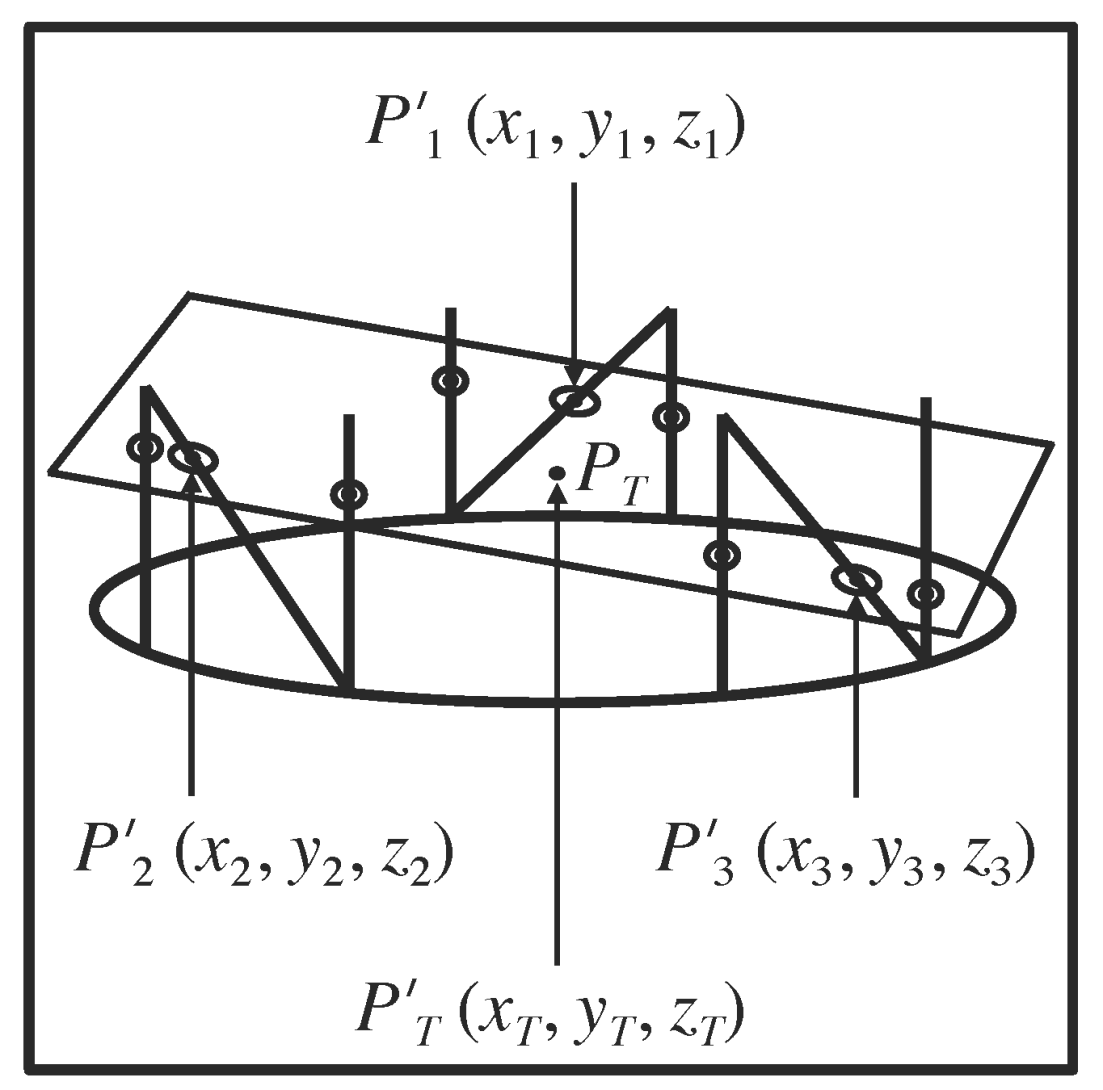
Representation of the Tomographic Section in the Three-Dimensional Coordinate System of the Stereotactic Frame The quadrilateral represents the tomographic section. The large oval depicts the circular base of the stereotactic frame (in perspective). The vertical and diagonal lines that are attached to the large oval represent the nine rods. The centers of the six fiducial circles and the three fiducial ellipses that are created in the tomographic image by these nine rods are shown as points that lie in the tomographic section. The tomographic section intersects the long axes of the three diagonal rods at the points ${P}'_{1}$, ${P}'_{2}$, and ${P}'_{3}$ that coincide with the respective centers $P_{1}$, $P_{2}$, and $P_{3}$ of the three ellipses (Figure 6). The $\left(x_{1},y_{1},z_{1}\right)$, $\left(x_{2},y_{2},z_{2}\right)$, and $\left(x_{3},y_{3},z_{3}\right)$ coordinates of the respective points of intersection ${P}'_{1}$, ${P}'_{2}$, and ${P}'_{3}$ are calculated in the three-dimensional coordinate system of the stereotactic frame using Equations 1 and 2. Because these three points determine the spatial orientation of a plane in three-dimensional space, the spatial orientation of the tomographic section is determined with respect to the stereotactic frame. The target point ${P}'_{T}$ lies in the tomographic section. The $\left(x_{T},y_{T},z_{T}\right)$ coordinates of this target point are calculated in the three-dimensional coordinate system of the stereotactic frame using Equation 5.

The attachment of three N-localizers to a stereotactic frame permits calculation of the $\left(x_{B_{1}},y_{B_{1}},z_{B_{1}}\right)$, $\left(x_{B_{2}},y_{B_{2}},z_{B_{2}}\right)$, and $\left(x_{B_{3}},y_{B_{3}},z_{B_{3}}\right)$ coordinates for the three respective points ${P}'_{B_{1}}$, ${P}'_{B_{2}}$, and ${P}'_{B_{3}}$ in the three-dimensional coordinate system of the stereotactic frame (Figure 4). These three points correspond respectively to the three analogous points $P_{B_{1}}$, $P_{B_{2}}$, and $P_{B_{3}}$ in the two-dimensional coordinate system of the tomographic image. In the following discussion, the symbols ${P}'_{1}$, ${P}'_{2}$, and ${P}'_{3}$ will be used as a shorthand notation for ${P}'_{B_{1}}$, ${P}'_{B_{2}}$, and ${P}'_{B_{3}}$. The symbols $P_{1}$, $P_{2}$, and $P_{3}$ will be used as a shorthand notation for $P_{B_{1}}$, $P_{B_{2}}$, and $P_{B_{3}}$.

**Figure 5:**
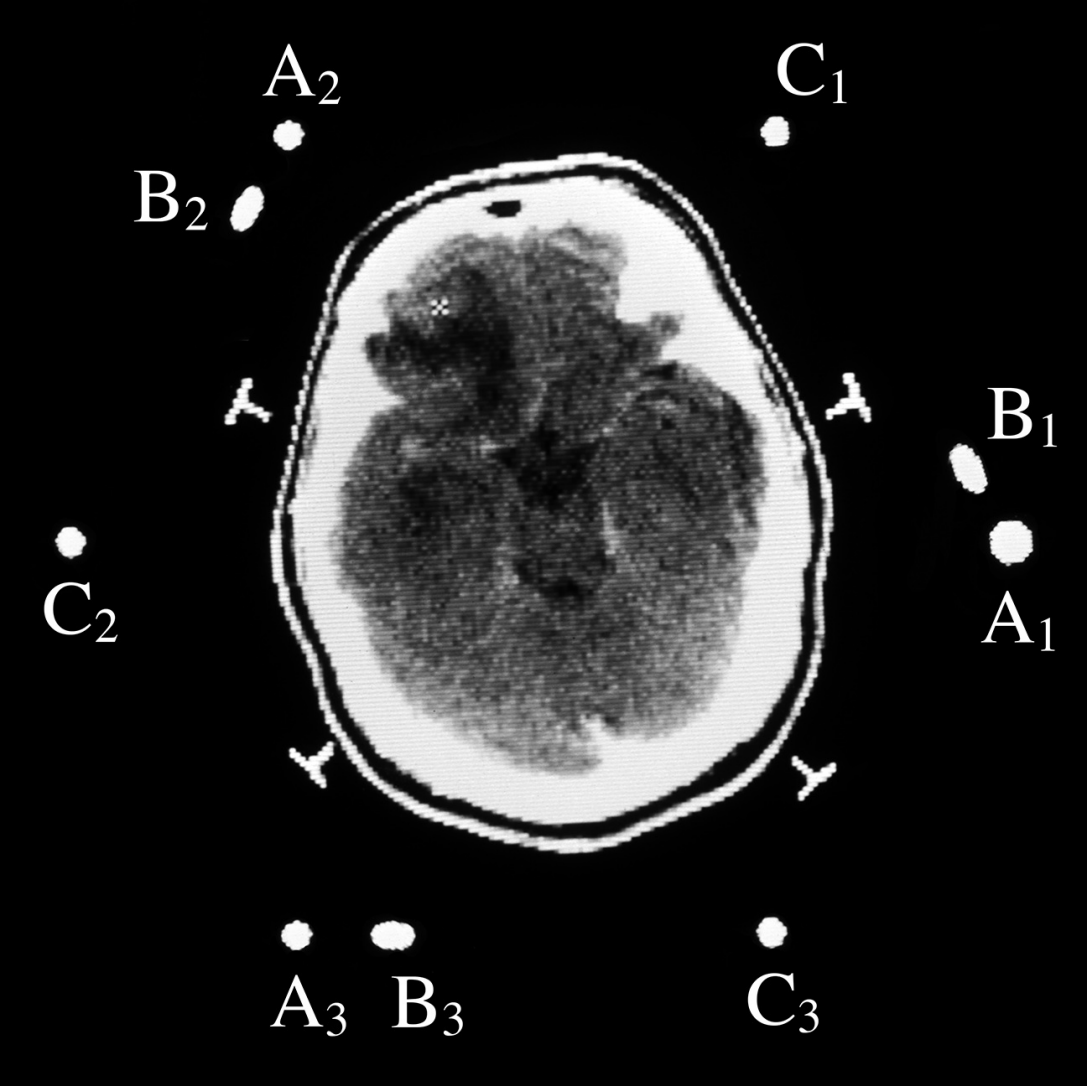
CT Image with Three Sets of Fiducials CT image of a patient to whom a BRW CT localizer frame (Integra Radionics Inc., Burlington, MA), which comprises three N-localizers, is attached. The cross sections of the three N-localizers create three sets of fiducials $\left\{ \mathrm{A_1,B_1,C_1} \right\}$, $\left\{ \mathrm{A_2,B_2,C_2} \right\}$, and $\left\{ \mathrm{A_3,B_3,C_3} \right\}$ in the CT image. The cursor indicates the target point $P_T$. The large vertical rod $\mathrm{A_1}$ allows it to be unambiguously distinguished from the other vertical rods and provides a visual cue that this figure is rotated approximately 90 degrees clockwise relative to Figure 6 [6].

**Figure 6:**
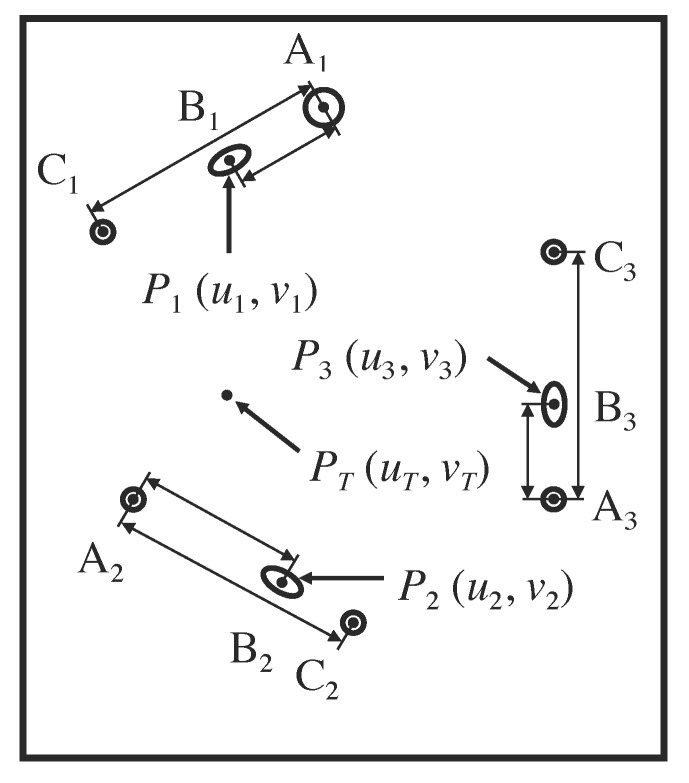
Representation of the Two-Dimensional Coordinate System of the Tomographic Image The cross sections of the three N-localizers create three sets of fiducials $\left \{ \mathrm{A_1,B_1,C_1} \right \}$, $\left \{ \mathrm{A_2,B_2,C_2} \right \}$, and $\left \{ \mathrm{A_3,B_3,C_3} \right \}$ in the tomographic image. Each set contains two circles and one ellipse that are collinear. For each set, the short double-ended arrows indicate the distance $d_{AB}$ between the centers of circle $\mathrm A$ and ellipse $\mathrm B$ and the long double-ended arrows indicate the distance $d_{AC}$ between the centers of circles $\mathrm A$ and $\mathrm C$. The centers $P_{1}$, $P_{2}$, and $P_{3}$ of the three ellipses coincide with the respective points of intersection ${P}'_{1}$, ${P}'_{2}$, and ${P}'_{3}$ of the long axes of the three diagonal rods with the tomographic section (Figure 4). The $\left(u_{1},v_{1}\right)$, $\left(u_{2},v_{2}\right)$, and $\left(u_{3},v_{3}\right)$ coordinates of the centers $P_{1}$, $P_{2}$, and $P_{3}$ correspond respectively to the $\left(x_{1},y_{1},z_{1}\right)$, $\left(x_{2},y_{2},z_{2}\right)$, and $\left(x_{3},y_{3},z_{3}\right)$ coordinates of the points of intersection ${P}'_{1}$, ${P}'_{2}$, and ${P}'_{3}$. The target point $P_{T}$ has $\left(u_{T},v_{T}\right)$ coordinates in the two-dimensional coordinate system of the tomographic image. The $\left(x_{T},y_{T},z_{T}\right)$ coordinates of the analogous target point ${P}'_{T}$ are calculated in the three-dimensional coordinate system of the stereotactic frame using Equation 5.

The three points ${P}'_{1}$, ${P}'_{2}$, and ${P}'_{3}$ lie on the three respective diagonal rods $\mathrm B_1$, $\mathrm B_2$, and $\mathrm B_3$ and have respective $\left(x,y,z\right)$ coordinates $\left(x_{1},y_{1},z_{1}\right)$, $\left(x_{2},y_{2},z_{2}\right)$, and $\left(x_{3},y_{3},z_{3}\right)$ in the three-dimensional coordinate system of the stereotactic frame (Figure 4). The analogous three points $P_{1}$, $P_{2}$, and $P_{3}$ lie at the centers of the three respective ellipses $\mathrm B_1$, $\mathrm B_2$, and $\mathrm B_3$ and have $\left(u,v\right)$ coordinates $\left(u_{1},v_{1}\right)$, $\left(u_{2},v_{2}\right)$, and $\left(u_{3},v_{3}\right)$ in the two-dimensional coordinate system of the tomographic image (Figures 5-6).

In order to facilitate calculation of the $\left(x_{T},y_{T},z_{T}\right)$ coordinates of the target point ${P}'_{T}$, it is convenient to project the $\left(u_{1},v_{1}\right)$, $\left(u_{2},v_{2}\right)$, and $\left(u_{3},v_{3}\right)$ coordinates of the three centers $P_{1}$, $P_{2}$, and $P_{3}$ of the ellipses onto the $w = 1$ plane in three-dimensional space by appending a third coordinate $w = 1$ to create $\left(u_{1},v_{1},1\right)$, $\left(u_{2},v_{2},1\right)$, and $\left(u_{3},v_{3},1\right)$ coordinates. The $w$-coordinate may be set arbitrarily to any non-zero value, e.g*.*, 1, so long as same value of $w$ is used for each of the three $w$-coordinates. The equations that are presented in the remainder of this article assume that a value of $w = 1$ has been used to project the $\left(u_{1},v_{1}\right)$, $\left(u_{2},v_{2}\right)$, and $\left(u_{3},v_{3}\right)$ coordinates. If a value of $w \neq 1$ were used instead of $w = 1$ to project these coordinates, the equations that are presented in the remainder of this article would no longer apply and would require revision so that the calculations that these equations describe may produce correct results.

Because three points determine the orientation of a plane in three-dimensional space, the three coordinates $\left(x_{1},y_{1},z_{1}\right)$, $\left(x_{2},y_{2},z_{2}\right)$, and $\left(x_{3},y_{3},z_{3}\right)$, together with the three coordinates $\left(u_{1},v_{1}\right)$, $\left(u_{2},v_{2}\right)$, and $\left(u_{3},v_{3}\right)$, determine the spatial orientation of the tomographic section with respect to the stereotactic frame. This spatial orientation or linear mapping is specified by the matrix elements $m_{11}$ through $m_{33}$ in the matrix equation

$$\begin{bmatrix} x_1 & y_1 & z_1 \\ x_2 & y_2 & z_2 \\ x_3 & y_3 & z_3 \end{bmatrix} = \begin{bmatrix} u_1 & v_1 & 1 \\ u_2 & v_2 & 1 \\ u_3 & v_3 & 1 \end{bmatrix} \begin{bmatrix} m_{11} & m_{12} & m_{13} \\ m_{21} & m_{22} & m_{23} \\ m_{31} & m_{32} & m_{33} \end{bmatrix}\;\;\;\;\;\;\;\;\;\;\left(3\right)$$

Equation 3 represents concisely a system of nine simultaneous linear equations that determine the spatial orientation of the tomographic section with respect to the stereotactic frame. This equation transforms the $\left(u_{1},v_{1}\right)$, $\left(u_{2},v_{2}\right)$, and $\left(u_{3},v_{3}\right)$ coordinates from the two-dimensional coordinate system of the tomographic image to create $\left(x_{1},y_{1},z_{1}\right)$, $\left(x_{2},y_{2},z_{2}\right)$, and $\left(x_{3},y_{3},z_{3}\right)$ coordinates in the three-dimensional coordinate system of the stereotactic frame.

In Equation 3, the matrix elements $x_1$ through $z_3$ as well as the matrix elements $u_1$ through $v_3$ are known. The matrix elements $m_{11}$ through $m_{33}$ are unknown; hence, Equation 3 may be inverted to solve for these unknown elements of the transformation matrix

$$\begin{bmatrix} m_{11} & m_{12} & m_{13} \\ m_{21} & m_{22} & m_{23} \\ m_{31} & m_{32} & m_{33} \end{bmatrix} = \begin{bmatrix} u_1 & v_1 & 1 \\ u_2 & v_2 & 1 \\ u_3 & v_3 & 1 \end{bmatrix}^{-1} \begin{bmatrix} x_1 & y_1 & z_1 \\ x_2 & y_2 & z_2 \\ x_3 & y_3 & z_3 \end{bmatrix}\;\;\;\;\;\;\;\;\;\;\left(4\right)$$

where the superscript operator -1 indicates the inverse of the matrix that contains the elements $u_1$ through $v_3$. The inverse of this matrix is guaranteed to exist so long as the $\left(u_{1},v_{1}\right)$, $\left(u_{2},v_{2}\right)$, and $\left(u_{3},v_{3}\right)$ coordinates of the centers of the three ellipses $\mathrm{B}_1$, $\mathrm{B}_2$, and $\mathrm{B}_3$ are not collinear. This non-collinearity is enforced by careful design of the stereotactic frame [7].

Once the transformation matrix elements $m_{11}$ through $m_{33}$ are known, the $\left(u_{T},v_{T}\right)$ coordinates of the target point $P_{T}$ may be transformed from the two-dimensional coordinate system of the tomographic image to the three-dimensional coordinate system of the stereotactic frame to obtain the $\left(x_{T},y_{T},z_{T}\right)$ coordinates of the analogous target point ${P}'_{T}$

$$\begin{bmatrix}x_{T}\;y_{T}\;z_{T}\end{bmatrix} = \begin{bmatrix}u_{T}\;v_{T}\;1\end{bmatrix} \begin{bmatrix} m_{11} & m_{12} & m_{13} \\ m_{21} & m_{22} & m_{23} \\ m_{31} & m_{32} & m_{33} \end{bmatrix}\;\;\;\;\;\;\;\;\;\;\left(5\right)$$

## Materials and methods

Equation 5 has been used for the past 37 years to calculate the $\left(x_{T},y_{T},z_{T}\right)$ coordinates of the target point ${P}'_{T}$ in the three-dimensional coordinate system of the stereotactic frame [2, 8]. This equation applies to only three N-localizers; however, some applications of the N-localizer have incorporated four N-localizers [9-13] that produce four sets of fiducials in a tomographic image. 

**Figure 7:**
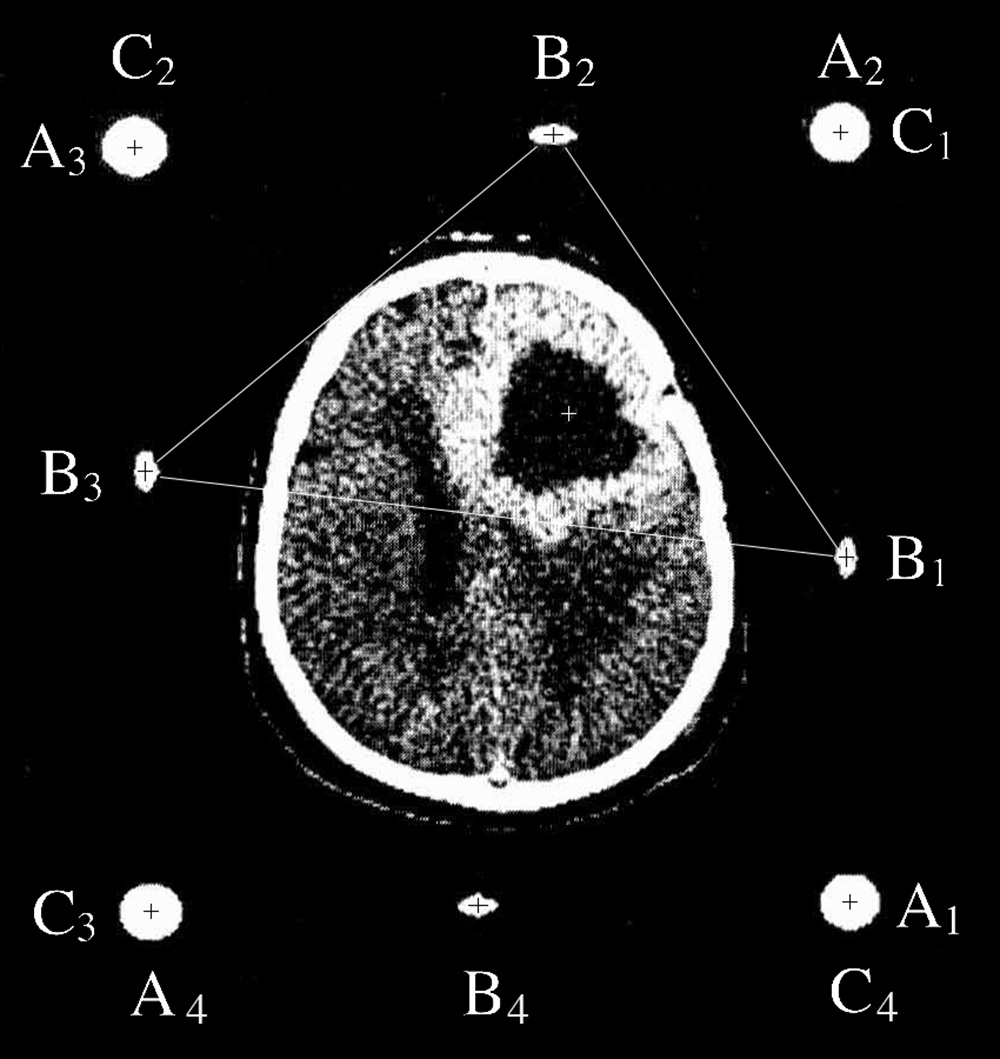
CT Image with Four Sets of Fiducials Four N-localizers create four sets of fiducials $\left\{ \mathrm{A_1,B_1,C_1} \right\}$, $\left\{ \mathrm{A_2,B_2,C_2} \right\}$, $\left\{ \mathrm{A_3,B_3,C_3} \right\}$, and $\left\{ \mathrm{A_4,B_4,C_4} \right\}$. The N-localizers are merged end-to-end such that $\mathrm{A_1=C_4}$, $\mathrm{A_2=C_1}$, $\mathrm{A_3=C_2}$, and $\mathrm{A_4=C_3}$. The black cross hairs indicate the centers of the fiducials and the white cross hairs indicate the target point $P_T$ that lies inside the triangle $\mathrm{B_1B_2B_3}$ (see text for explanation). Adapted from [9].

Four sets of fiducials are visible in the CT image of Figure 7. The transformation or linear mapping from the two-dimensional coordinate system of this tomographic image into the three-dimensional coordinate system of the stereotactic frame may be represented as

$$\begin{bmatrix} x_1 & y_1 & z_1 \\ x_2 & y_2 & z_2 \\ x_3 & y_3 & z_3 \\ x_4 & y_4 & z_4 \end{bmatrix} = \begin{bmatrix} u_1 & v_1 & 1 \\ u_2 & v_2 & 1 \\ u_3 & v_3 & 1 \\ u_4 & v_4 & 1 \end{bmatrix} \begin{bmatrix} m_{11} & m_{12} & m_{13} \\ m_{21} & m_{22} & m_{23} \\ m_{31} & m_{32} & m_{33} \end{bmatrix}\;\;\;\;\;\;\;\;\;\;\left(6\right)$$

An important distinction between Equations 3 and 6 is that Equation 3 may be inverted via Equation 4 to solve for the transformation matrix elements $m_{11}$ through $m_{33}$ whereas Equation 6 may not be inverted to obtain these transformation matrix elements because Equation 6 includes non-square matrices [7].

One solution to this problem is to ignore one of the four sets of fiducials and to use the remaining three sets of fiducials for Equations 3 and 4. This solution raises a question concerning which set of fiducials to ignore. One approach to ignoring a set of fiducials is to attempt to minimize errors by choosing the three fiducial points $\mathrm{B_i}$ that form a triangle that encloses the target point $P_T$ [9]. For example, in Figure 7, the target point lies within the triangle $\mathrm{B_1B_2B_3}$, so fiducial $\mathrm{B_4}$ would be ignored for application of Equation 4. Although this approach aims to minimize errors, it requires that important data, *i.e.*, one set of fiducials, be ignored.

It is possible to minimize error via the method of least squares [14] without ignoring any of the fiducials. The least-squares method applies to three or more sets of fiducials. The equations that are required for least-squares minimization are obtained by first expanding the matrix multiplication of Equation 6 and expressing the result for the matrix elements $x_i$, $y_i$, and $z_i$

$$\begin{matrix} x_i = u_i m_{11} + v_i m_{21} + m_{31} \\ y_i = u_i m_{12} + v_i m_{22} + m_{32} \\ z_i = u_i m_{13} + v_i m_{23} + m_{33} \end{matrix}\;\;\;\;\;\;\;\;\;\;\left(7\right)$$

where the subscript $i$ designates the matrix row. In the presence of error, Equation 7 may be rearranged to express the errors in $x_i$, $y_i$, and $z_i$ as $\delta x_i$, $\delta y_i$, and $\delta z_i$, respectively

$$\begin{matrix} \delta x_i = x_i - u_i m_{11} - v_i m_{21} - m_{31} \\ \delta y_i = y_i - u_i m_{12} - v_i m_{22} - m_{32} \\ \delta z_i = z_i - u_i m_{13} - v_i m_{23} - m_{33} \end{matrix}\;\;\;\;\;\;\;\;\;\;\left(8\right)$$

In order to minimize these errors via the method of least squares, the values of $\delta x_i$, $\delta y_i$, and $\delta z_i$ are squared to obtain the error functions $E_x$, $E_y$, and $E_z$

$$\begin{matrix} E_x \left(m_{11}, m_{21}, m_{31}\right) = \sum{ \left(x_i - u_i m_{11} - v_i m_{21} - m_{31} \right)^2} \\ E_y \left(m_{12}, m_{22}, m_{32}\right) = \sum{ \left(y_i - u_i m_{12} - v_i m_{22} - m_{32} \right)^2} \\ E_z \left(m_{13}, m_{23}, m_{33}\right) = \sum{ \left(z_i - u_i m_{13} - v_i m_{23} - m_{33} \right)^2} \end{matrix}\;\;\;\;\;\;\;\;\;\;\left(9\right)$$

The following discussion illustrates minimization of the error function $E_x$; minimization of the error functions $E_y$ and $E_z$ is performed in an analogous manner. At the minimum of a function, all of the derivatives are equal to zero. Evaluating the derivatives $\partial E_x / \partial m_{11}$, $\partial E_x / \partial m_{21}$, and $\partial E_x / \partial m_{31}$ and setting the resulting expressions for these derivatives to zero yields

$$\begin{matrix} \partial E_x / \partial m_{11} = \sum{ 2 \left(x_i - u_i m_{11} - v_i m_{21} - m_{31} \right) u_i } = 0 \\ \partial E_x / \partial m_{21} = \sum{ 2 \left(x_i - u_i m_{11} - v_i m_{21} - m_{31} \right) v_i } = 0 \\ \partial E_x / \partial m_{31} = \sum{ 2 \left(x_i - u_i m_{11} - v_i m_{21} - m_{31} \right) } = 0 \end{matrix}\;\;\;\;\;\;\;\;\;\;\left(10\right)$$

Simplifying and rearranging the above equations for the derivatives yields a system of three simultaneous linear equations of the three unknowns $m_{11}$, $m_{21}$, and $m_{31}$

$$\begin{matrix} m_{11} \sum{{u_i}^2} + m_{21} \sum {u_i v_i} + m_{31} \sum{ u_i} = \sum{u_i x_i} \\ m_{11} \sum{u_i v_i} + m_{21} \sum{{v_i}^2} + m_{31} \sum{ v_i} = \sum{v_i x_i} \\ m_{11} \sum{u_i} + m_{21} \sum{v_i} + m_{31} n = \sum{x_i} \end{matrix}\;\;\;\;\;\;\;\;\;\;\left(11\right)$$

where $n$ is the number of sets of fiducials; in the case of Equation 6, $n = 4$. These simultaneous linear equations may be solved using Cramer's rule [15] or perferably Gauss elimination [16] to yield the matrix elements $m_{11}$, $m_{21}$, and $m_{31}$ that minimize the error function $E_x$. The error functions $E_y$ and $E_z$ are minimized in a similar manner to obtain the matrix elements $m_{12}$, $m_{22}$, and $m_{32}$ and the matrix elements $m_{13}$, $m_{23}$, and $m_{33}$, respectively.

Once the elements of the transformation matrix have been calculated as discussed above, the transformation matrix may be used as shown in Equation 5 to transform the $\left(u_{T},v_{T}\right)$ coordinates of the target point $P_{T}$ from the two-dimensional coordinate system of the tomographic image to the three-dimensional coordinate system of the stereotactic frame to obtain the $\left(x_{T},y_{T},z_{T}\right)$ coordinates of the analogous target point ${P}'_{T}$.

The accuracy of the calculation of the transformation matrix elements, and hence, the accuracy of the transformation of the $\left(u_{T},v_{T}\right)$ coordinates, is indicated by the correlation coefficient $r_{xyz}$ that is a measure of how well the $\left(x_i,y_i,z_i\right)$ coordinates fit the plane equation

$$ax + by + cz = 0\;\;\;\;\;\;\;\;\;\;\left(12 \right )$$

This correlation coefficient may be obtained by first calculating the three linear correlation coefficients $r_{xz}$, $r_{yz}$, and $r_{xy}$ in the manner that is shown below for $r_{xy}$ [17]

$$r_{xy} = \frac{n \sum {x_i y_i} - \sum {x_i} \sum {y_i}}{\sqrt{ n \sum {x_i}^2 - \left( \sum x_i\right )^2 } \sqrt{ n\sum {y_i}^2 - \left( \sum y_i\right )^2}}\;\;\;\;\;\;\;\;\;\;\left(13\right)$$

then combining these linear correlation coefficients to obtain a coefficient of multiple correlation [18]

$$r_{xyz} = \sqrt{\frac{{r_{xz}}^2 + {r_{yz}}^2 - 2 r_{xz} r_{yz} r_{xy}}{1 - {r_{xy}}^2}}\;\;\;\;\;\;\;\;\;\;\left(14\right)$$

## Results

Figure 7 is a CT image wherein four N-localizers have produced four sets of fiducials. A cursor was centered over the cross hairs for each of the eight fiducials and for the target point $P_T$ in order to read the $\left(u,v\right)$ coordinates of the fiducials and the target point. These coordinates are shown in Table 1.

**Table 1:** The $\left(u,v\right)$ Coordinates of Fiducials and Target Point $P_T$ from Figure 7 The $\left(u,v\right)$ coordinates of the fiducials and the target point $P_T$ were measured by centering a cursor over the cross hairs in the CT image of Figure 7. The position of the reference origin of the CT image and the units of measurement of $u_i$ and $v_i$ (millimeters, pixels, etc.) are irrelevant, so long as the same reference origin and units of measurement are used to measure each $u_i$ and $v_i$. Also, independent of whether the $u$-coordinates are measured in the horizontal direction and the $v$-coordinates are measured in the vertical direction, or *vice versa*, Equation 5 will calculate the same $\left(x_T,y_T,z_T\right)$ coordinates for the analogous target point ${P}'_T$.

Cross Hair	$u$	$v$
$\mathrm{A_1 = C_4}$	2.409	2.553
$\mathrm{B_1}$	2.397	1.577
$\mathrm{A_2=C_1}$	2.382	0.374
$\mathrm{B_2}$	1.567	0.382
$\mathrm{A_3=C_2}$	0.380	0.418
$\mathrm{B_3}$	0.411	1.336
$\mathrm{A_4=C_3}$	0.429	2.581
$\mathrm{B_4}$	1.354	2.566
$P_T$	1.612	1.171

The $\left(u_i,v_i\right)$ coordinates for all four fiducials $\mathrm B_1$, $\mathrm B_2$, $\mathrm B_3$, and $\mathrm B_4$ from Table 1 were used to construct a transformation matrix by solving Equation 11, assuming the stereotactic frame to be a cube whose sides are 30 cm long (see Appendix 1 for details). Then this transformation matrix was used to transform the $\left(u_T,v_T\right)$ coordinates of the target point $P_T$ that are shown in Table 1 into the $\left(x_T,y_T,z_T\right)$ coordinates of the analogous target point ${P}'_{T\left(4\right)}$ that are shown in Table 2. The correlation coefficient $r_{xyz} = 0.99998$ was calculated to indicate the accuracy of the transformation.

In order to assess the effect of ignoring one set of fiducials upon the accuracy of the transformation, a different transformation matrix was calculated via Equation 4 using the $\left(u_i,v_i\right)$ coordinates from Table 1 for each of the four combinations of fiducials $\mathrm{B_1B_2B_3}$, $\mathrm{B_2B_3B_4}$, $\mathrm{B_3B_4B_1}$, and $\mathrm{B_4B_1B_2}$. Then these four different transformation matrices were used to transform the $\left(u_T,v_T\right)$ coordinates of the target point $P_T$ that are shown in Table 1 into $\left(x_T,y_T,z_T\right)$ coordinates for the four different target points ${P}'_{T\left(3\right)}$ that are shown in Table 2. Also, the Pythagorean distance $d$, which represesents the transformation error, was calculated between each of these four target points ${P}'_{T\left(3\right)}$ and the target point ${P}'_{T\left(4\right)}$. The mean transformation error is $0.535$ mm and the standard deviation is $0.270$ mm.

**Table 2:** The $\left(x,y,z\right)$ Coordinates of the Target Point ${P}'_T$ Calculated from Figure 7 The $\left(x,y,z\right)$ coordinates in centimeters for the target point ${P}'_{T\left(4\right)}$ were calculated using all four fiducials $\mathrm B_1$, $\mathrm B_2$, $\mathrm B_3$, and $\mathrm B_4$ from Figure 7. Also, the $\left(x,y,z\right)$ coordinates in centimeters for the four different target points ${P}'_{T\left(3\right)}$ were calculated using all four combinations of three fiducials. The Pythagorean distance $d$ from ${P}'_{T\left(4\right)}$ to each ${P}'_{T\left(3\right)}$ is indicated in millimeters.

Target Point and Fiducials	$x$ (cm)	$y$ (cm)	$z$ (cm)	$d$ (mm)
${P}'_{T\left(4\right)}\;\;\mathrm{B_1B_2B_3B_4}$	3.246	4.178	2.106	
${P}'_{T\left(3\right)}\;\;\mathrm{B_1B_2B_3}$	3.235	4.199	2.105	0.237
${P}'_{T\left(3\right)}\;\;\mathrm{B_2B_3B_4}$	3.278	4.120	2.107	0.662
${P}'_{T\left(3\right)}\;\;\mathrm{B_3B_4B_1}$	3.206	4.252	2.103	0.842
${P}'_{T\left(3\right)}\;\;\mathrm{B_4B_1B_2}$	3.265	4.143	2.107	0.398

**Figure 8:**
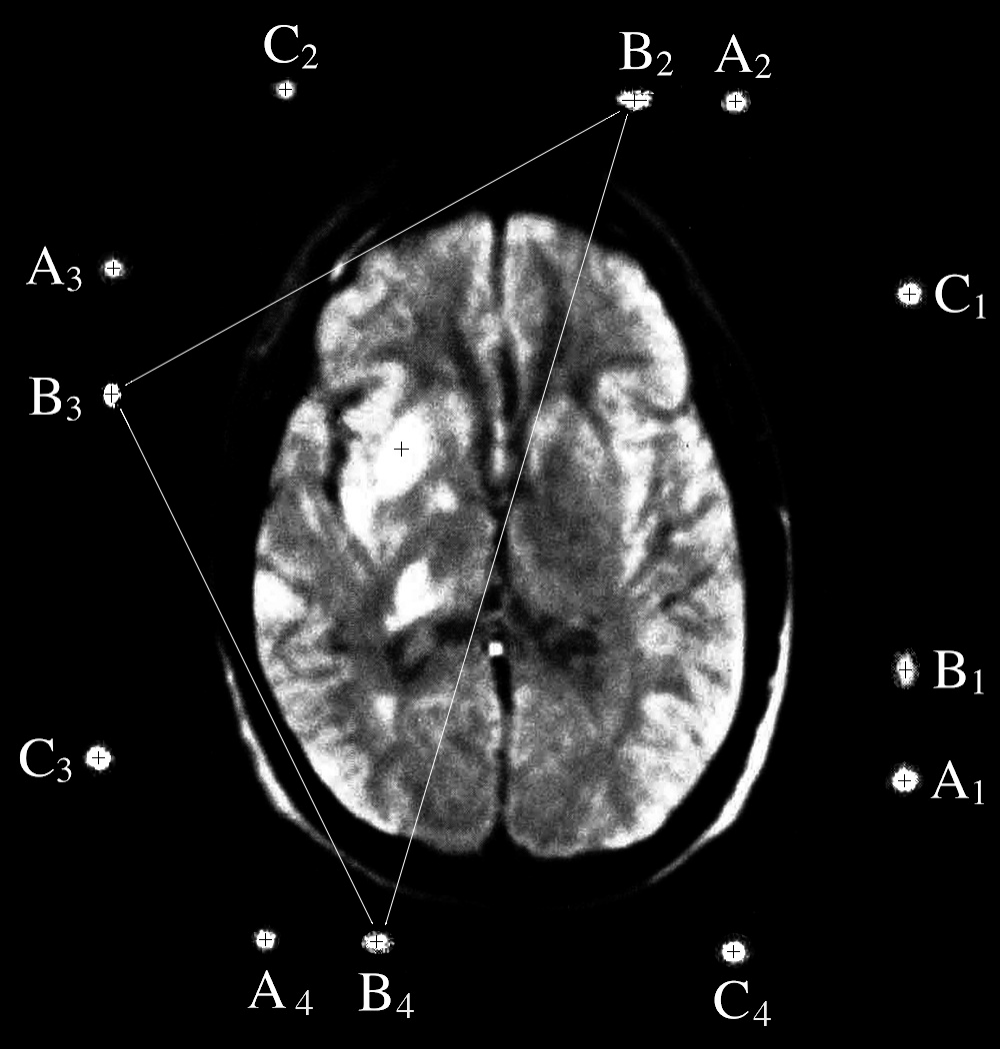
MR Image with Four Sets of Fiducials MR image of a patient to whom a first-generation BRW MR localizer frame (Radionics Inc., Burlington, MA) is attached. Four N-localizers create four sets of fiducials $\left\{ \mathrm{A_1,B_1,C_1} \right\}$, $\left\{ \mathrm{A_2,B_2,C_2} \right\}$, $\left\{ \mathrm{A_3,B_3,C_3} \right\}$, and $\left\{ \mathrm{A_4,B_4,C_4} \right\}$. The black cross hairs indicate the centers of the fiducials and the target point $P_T$. Adapted from [11].

Figure 8 is a MR image wherein four N-localizers have produced four sets of fiducials. A cursor was centered over the cross hairs for each of the 12 fiducials and for the target point $P_T$ in order to read the $\left(u,v\right)$ coordinates of the fiducials and the target point. These coordinates are shown in Table 3.

**Table 3:** The $\left(u,v\right)$ Coordinates of Fiducials and Target Point $P_T$ from Figure 8 The $\left(u,v\right)$ coordinates of the fiducials and the target point $P_T$ were measured by centering a cursor over the cross hairs in the MR image of Figure 8.

Cross Hair	$u$	$v$
$\mathrm{A_1}$	3.014	2.604
$\mathrm{B_1}$	3.018	2.234
$\mathrm{C_1}$	3.030	0.981
$\mathrm{A_2}$	2.451	0.338
$\mathrm{B_2}$	2.114	0.334
$\mathrm{C_2}$	0.950	0.298
$\mathrm{A_3}$	0.378	0.894
$\mathrm{B_3}$	0.371	1.314
$\mathrm{C_3}$	0.328	2.528
$\mathrm{A_4}$	0.884	3.134
$\mathrm{B_4}$	1.254	3.141
$\mathrm{C_4}$	2.444	3.174
$P_T$	1.337	1.499

The $\left(u_i,v_i\right)$ coordinates for all four fiducials $\mathrm B_1$, $\mathrm B_2$, $\mathrm B_3$, and $\mathrm B_4$ from Table 3 were used to construct a transformation matrix by solving Equation 11, assuming the stereotactic frame to be a cube whose sides are 30 cm long (see Appendix 2 for details). Then this transformation matrix was used to transform the $\left(u_T,v_T\right)$ coordinates of the target point $P_T$ that are shown in Table 3 into the $\left(x_T,y_T,z_T\right)$ coordinates of the analogous target point ${P}'_{T\left(4\right)}$ that are shown in Table 4. The correlation coefficient $r_{xyz} = 0.88977$ was calculated to indicate the accuracy of the transformation.

In order to assess the effect of ignoring one set of fiducials upon the accuracy of the transformation, a different transformation matrix was calculated via Equation 4 using the $\left(u_i,v_i\right)$ coordinates from Table 3 for each of the four combinations of fiducials $\mathrm{B_1B_2B_3}$, $\mathrm{B_2B_3B_4}$, $\mathrm{B_3B_4B_1}$, and $\mathrm{B_4B_1B_2}$. Then these four different transformation matrices were used to transform the $\left(u_T,v_T\right)$ coordinates of the target point $P_T$ that are shown in Table 3 into $\left(x_T,y_T,z_T\right)$ coordinates for the four different target points ${P}'_{T\left(3\right)}$ that are shown in Table 4. Also, the Pythagorean distance $d$, which represents the transformation error, was calculated between each of these four target points ${P}'_{T\left(3\right)}$ and the target point ${P}'_{T\left(4\right)}$. The mean transformation error is $2.139$ mm and the standard deviation is $1.061$ mm.

**Table 4:** The $\left(x,y,z\right)$ Coordinates of the Target Point ${P}'_T$ Calculated from Figure 8 The $\left(x,y,z\right)$ coordinates in centimeters for the target point ${P}'_{T\left(4\right)}$ were calculated using all four fiducials $\mathrm B_1$, $\mathrm B_2$, $\mathrm B_3$, and $\mathrm B_4$ from Figure 8. Also, the $\left(x,y,z\right)$ coordinates in centimeters for the four different target points ${P}'_{T\left(3\right)}$ were calculated using all four combinations of three fiducials. The Pythagorean distance $d$ from ${P}'_{T\left(4\right)}$ to each ${P}'_{T\left(3\right)}$ is indicated in millimeters.

Target Point and Fiducials	$x$ (cm)	$y$ (cm)	$z$ (cm)	$d$ (mm)
${P}'_{T\left(4\right)}\;\;\mathrm{B_1B_2B_3B_4}$	-3.760	2.988	7.791	
${P}'_{T\left(3\right)}\;\;\mathrm{B_1B_2B_3}$	-3.858	3.010	7.647	1.756
${P}'_{T\left(3\right)}\;\;\mathrm{B_2B_3B_4}$	-3.711	2.977	7.863	0.878
${P}'_{T\left(3\right)}\;\;\mathrm{B_3B_4B_1}$	-3.904	3.020	7.578	2.591
${P}'_{T\left(3\right)}\;\;\mathrm{B_4B_1B_2}$	-3.575	2.946	8.065	3.333

For the CT image of Figure 7, the correlation coefficient $r_{xyz} = 0.99998$ and the mean transformation error of $0.535$ mm indicate that only a small amount of error is present in the CT image. A possible source of this error is the fact that the $\left(u,v\right)$ coordinates of the centers of the fiducials were recorded manually using a cursor and hence these coordinates are accurate to only the nearest pixel. In practice, this source of error is greatly reduced by computer software that calculates the center of each fiducial at sub-pixel precision instead of relying on a human to identify the center of the fiducial manually.

The attempt to minimize the transformation error by ignoring one set of fiducials [9] does diminish the error, as can be seen from Figure 7 and Table 2. Assuming that ${P}'_{T\left(4\right)}$, which was calculated using all four sets of fiducials, is the most accurate target point, it is evident that the Pythagorean distance $d$ from ${P}'_{T\left(4\right)}$ to ${P}'_{T\left(3\right)}$ varies with the position of $P_T$ relative to the triangle that is formed by the three $\mathrm{B_i}$ that are used for application of Equation 4. Specifically, the distance $d$ increases as the position of $P_T$ progresses from well inside triangle $\mathrm{B_1B_2B_3}$ to marginally inside triangle $\mathrm{B_4B_1B_2}$ to marginally outside triangle $\mathrm{B_2B_3B_4}$ to well outside triangle $\mathrm{B_3B_4B_1}$. Figure 8 and Table 4 show a similar trend of increasing Pythagorean distance $d$ from ${P}'_{T\left(4\right)}$ to ${P}'_{T\left(3\right)}$ as the position of $P_T$ progresses from well inside triangle $\mathrm{B_2B_3B_4}$ to marginally inside triangle $\mathrm{B_1B_2B_3}$ to marginally outside triangle $\mathrm{B_3B_4B_1}$ to well outside triangle $\mathrm{B_4B_1B_2}$. Because the Pythagorean distance $d$ represents the transformation error, these trends demonstrate that the transformation error may be minimized to some extent by choosing the three fiducials $\mathrm{B_i}$ that form a triangle that encloses the target point $P_T$. However, choosing three of the four fiducials $\mathrm{B_i}$ ignores one set of fiducials and, hence, requires that important data be discarded, whereas least-squares minimization uses all four sets of fiducials and thus discards no data.

For the MR image of Figure 8, the correlation coefficient $r_{xyz} = 0.88977$ and the mean transformation error of $2.139$ mm indicate that substantially more error is present in the MR image of Figure 8 than in the CT image of Figure 7. A likely source of this error is nonlinear distortion of the MR image that may be caused by metallic elements of the stereotactic frame, inhomogeneity and temporal fluctuation of the magnetic field, and metallic equipment near the MR scanner [11, 19-22].

In view of the N-localizer's requirement for linearity, the susceptibility of MR to nonlinear distortion can potentially degrade the accuracy of MR-guided stereotactic surgery [7]. In the absence of nonlinear distortion, the centers of the two circles $\mathrm{A_i}$ and $\mathrm{C_i}$ and the ellipse $\mathrm{B_i}$ are expected to be collinear, as shown in Figure 2b and Figure 6. Hence, it has been suggested that the linearity of an MR image may be checked by calculating a correlation coefficient $r_{uv\left(i\right)}$ for each of the four sets of fiducials $\left \{ \mathrm{A_i,B_i,C_i} \right \}$ via Equation 13 using the $\left(u,v\right)$ coordinates of the centers of the three fiducials $\mathrm{A_i}$, $\mathrm{B_i}$, and $\mathrm{C_i}$ [20]. However, this test for linearity is sensitive to nonlinear distortion only if the distortion causes the center of fiducial $\mathrm{B_i}$ to move perpendicularly to the line that connects the centers of fiducials $\mathrm{A_i}$ and $\mathrm{C_i}$. This test for linearity is insensitive to the case where the distortion causes the center of fiducial $\mathrm{B_i}$ to move along the line that connects the centers of fiducials $\mathrm{A_i}$ and $\mathrm{C_i}$ because in this case the value of the correlation coefficient $r_{uv\left(i\right)}$ does not change.

On the other hand, the correlation coefficient $r_{xyz}$ that is calculated via Equation 14 is sensitive to any displacement of the centers of fiducials $\mathrm{B_i}$ relative to the centers of fiducials $\mathrm{A_i}$ and $\mathrm{C_i}$. Moreover, the correlation coefficient $r_{xyz}$ is sensitive to the displacement of the center of any fiducial relative to the center of any other fiducial. Such a displacement alters the calculation of the $\left(x_i,y_i,z_i\right)$ coordinates of one or more of the four ${P}'_i$ via Equations 1 and 2, and these altered coordinates affect the correlation coefficient $r_{xyz}$. The robustness and usefulness of the correlation coefficient $r_{xyz}$ underscore the superiority of least-squares minimization compared to ignoring one of the four sets of fiducials [9].

Table 5 shows that the four correlation coefficients $r_{uv\left(i\right)}$ are insensitive to the nonlinear distortion that is present in the MR image of Figure 8. The correlation coefficients $r_{uv\left(i\right)}$ that are calculated for each of the four sets of fiducials $\left \{ \mathrm{A_i,B_i,C_i} \right \}$ are substantially larger than the correlation coefficient $r_{xyz}$ that is calculated using the four fiducials $\mathrm B_1$, $\mathrm B_2$, $\mathrm B_3$, and $\mathrm B_4$. This disparity between $r_{xyz}$ and the four $r_{uv\left(i\right)}$ suggests that the distortion in the MR image of Figure 8 either causes the centers of the four fiducials $\mathrm{B_i}$ to move along the lines that connect the centers of fiducials $\mathrm{A_i}$ and $\mathrm{C_i}$, or causes the four sets of fiducials $\left \{ \mathrm{A_i,B_i,C_i} \right \}$ to move relative to one another in a manner that does not affect the collinear relationship within each set of fiducials $\left \{ \mathrm{A_i,B_i,C_i} \right \}$.

**Table 5:** Correlation Coefficients Calculated from the Fiducials of Figure 8 The correlation coefficients that are calculated from the $\left(u,v\right)$ coordinates of the fiducials in the MR image of Figure 8 indicate that the correlation coefficients $r_{uv\left(i\right)}$ are insensitive to nonlinear distortion of this MR image, whereas the correlation coefficient $r_{xyz}$ is sensitive to that distortion.

Correlation Coefficient	Value
$r_{uv\left(1\right)}$	0.99973
$r_{uv\left(2\right)}$	0.99223
$r_{uv\left(3\right)}$	0.99276
$r_{uv\left(4\right)}$	0.99793
$r_{xyz}$	0.88977

Four or more N-localizers require the solution of Equation 11 instead of using Equation 4 to calculate the transformation matrix. For three N-localizers, either approach may be used to calculate the transformation matrix but for three N-localizers there is no advantage to solving Equation 11. The correlation coefficient $r_{xyz}$ that is calculated via Equation 14 is a valid statistical measure of the accuracy of the transformation for only four or more N-localizers. For three N-localizers, this correlation coefficient equals $1.0$ because three points determine the orientation of a plane in three-dimensional space.

## Discussion

Magnetic resonance (MR) imaging differs from computed tomography (CT) imaging in the manner by which the images are obtained. CT obtains a volume of individual tomographic images of the patient by changing the position of the scanner bed between successive tomographic scans and, hence, is susceptible to errors in scanner bed positioning. MR obtains a volume image of the patient by applying magnetic field gradients [23] and, therefore, does not require changing the position of the scanner bed. Indeed, MR may obtain a volume image of the patient directly without obtaining a series of planar, tomographic scans. Because MR is not susceptible to errors in scanner bed positioning, the spatial accuracy of a MR volume image ought to be greater than the spatial accuracy of a volume of successive CT images, provided that the patient does not move during the imaging procedure.

A volume image comprises individual volume elements, or voxels, that are identified via their $\left(u, v, w\right)$ coordinates in the same manner that the individual picture elements, or pixels, from a planar, tomographic image are identified via their $\left(u, v\right)$ coordinates. A planar section of these voxels is a subset of the voxels wherein one of the $\left(u, v, w\right)$ coordinates is held constant. An axial plane has constant $w$ and varying $\left(u, v\right)$ coordinates. A sagittal plane has constant $u$ and varying $\left(v, w\right)$ coordinates. A coronal plane has constant $v$ and varying $\left(u, w\right)$ coordinates. In this context, the term "planar section" or "plane" designates an axial, sagittal or coronal plane, i.e., a subset of the voxels that one volume image comprises. Such a plane is to be distinguished from a tomographic image that comprises a set of pixels that are obtained via one planar, tomographic scan.

**Figure 9:**
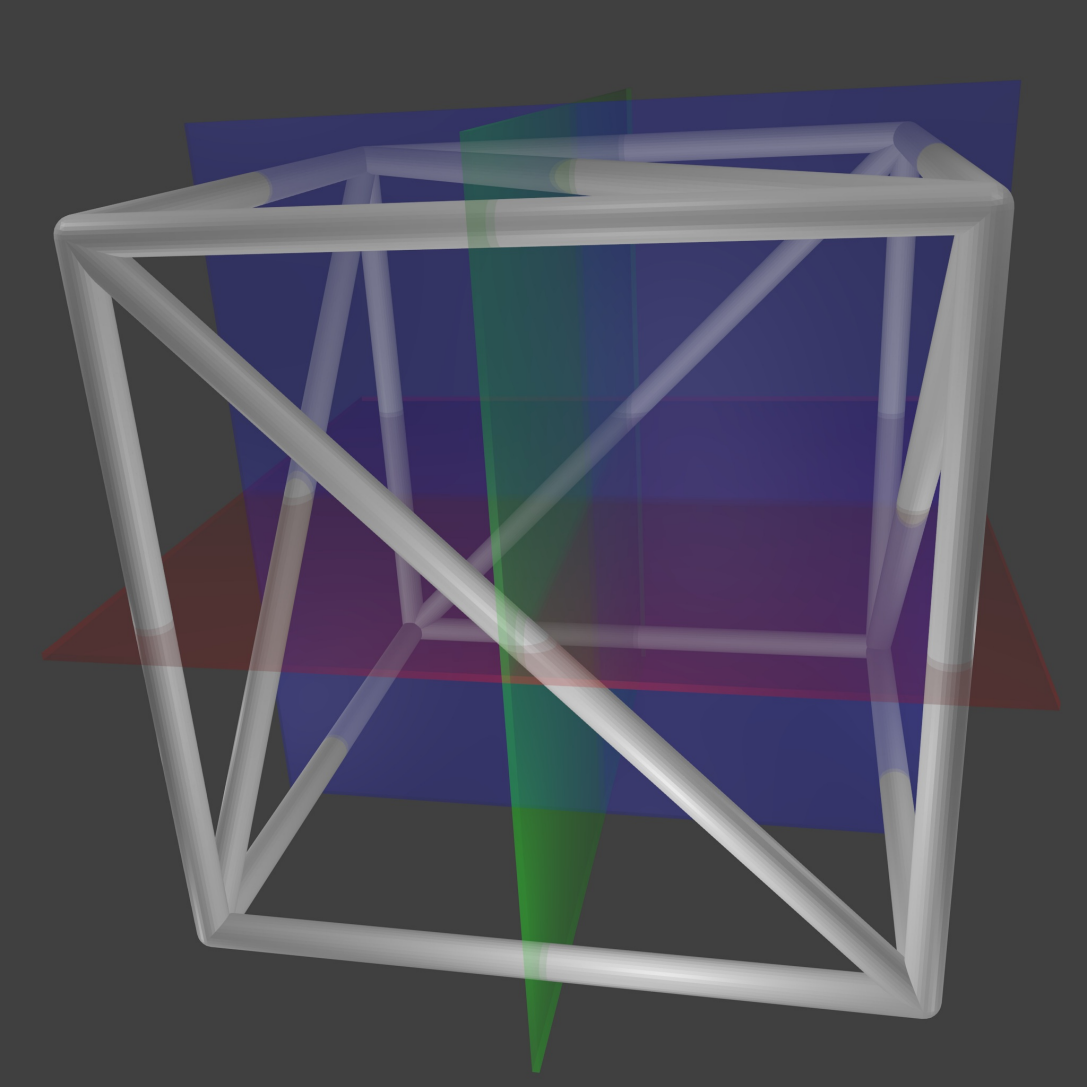
MR Localizer Frame and Axial, Sagittal and Coronal Planes A MR localizer frame comprises five N-localizers that subtend the anterior, posterior, left, right and superior faces of a cube. An axial plane (red) intersects the MR localizer frame at four N-localizers: anterior, posterior, left and right. A sagittal plane (green) intersects the MR localizer frame at three N-localizers: anterior, posterior and superior. A coronal plane (blue) intersects the MR localizer frame at three N-localizers: left, right and superior.

A MR localizer frame differs from a CT localizer frame in that the MR localizer frame is designed to create fiducials in sagittal and coronal planes in addition to axial planes [24]. A MR localizer frame comprises five N-localizers that subtend the anterior, posterior, left, right, and superior faces of a cube that encloses the patient's head (Figure 9).

In a manner analogous to Equation 3, the spatial orientation of MR voxel data may be determined with respect to the stereotactic frame. The equation that applies to these voxel data requires four $\left(x, y, z\right)$ coordinates $\left(x_{1},y_{1},z_{1}\right)$, $\left(x_{2},y_{2},z_{2}\right)$, $\left(x_{3},y_{3},z_{3}\right)$, and $\left(x_{4},y_{4},z_{4}\right)$ from the three-dimensional coordinate system of the stereotactic frame. This equation also requires four $\left(u, v, w\right)$ coordinates $\left(u_{1},v_{1},w_{1}\right)$, $\left(u_{2},v_{2},w_{2}\right)$, $\left(u_{3},v_{3},w_{3}\right)$, and $\left(u_{4},v_{4},w_{4}\right)$ from the three-dimensional coordinate system of the voxel data. The $\left(u, v, w\right)$ coordinates are the centers of ellipses $\mathrm{B_i}$ that are visualized in axial, sagittal or coronal planes of the voxel data, similar to the approach that is discussed for an axial tomographic section in Figure 6. The $\left(x, y, z\right)$ coordinates are calculated from the $\left(u, v, w\right)$ coordinates of the centers of circles $\mathrm{A_i}$ and $\mathrm{C_i}$ and ellipses $\mathrm{B_i}$ via Equation 1 or Equation 2.

The spatial orientation or linear mapping of the voxel data with respect to the stereotactic frame is specified using the matrix equation [25]

$$\begin{bmatrix} x_1 & y_1 & z_1 & 1 \\ x_2 & y_2 & z_2 & 1 \\ x_3 & y_3 & z_3 & 1 \\ x_4 & y_4 & z_4 & 1 \end{bmatrix} = \begin{bmatrix} u_1 & v_1 & w_1 & 1 \\ u_2 & v_2 & w_2 & 1 \\ u_3 & v_3 & w_3 & 1 \\ u_4 & v_4 & w_4 & 1 \end{bmatrix} \begin{bmatrix} m_{11} & m_{12} & m_{13} & 0 \\ m_{21} & m_{22} & m_{23} & 0 \\ m_{31} & m_{32} & m_{33} & 0 \\ m_{41} & m_{42} & m_{43} & 1 \end{bmatrix}\;\;\;\;\;\;\;\;\;\;\left(15\right)$$

Equation 15 represents concisely a system of 12 simultaneous linear equations that determine the spatial orientation of the voxel data with respect to the stereotactic frame. This equation transforms the $\left(u_{1},v_{1},w_{1}\right)$, $\left(u_{2},v_{2},w_{2}\right)$, $\left(u_{3},v_{3},w_{3}\right)$, and $\left(u_{4},v_{4},w_{4}\right)$ coordinates from the three-dimensional coordinate system of the voxel data to create $\left(x_{1},y_{1},z_{1}\right)$, $\left(x_{2},y_{2},z_{2}\right)$, $\left(x_{3},y_{3},z_{3}\right)$, and $\left(x_{4},y_{4},z_{4}\right)$ coordinates in the three-dimensional coordinate system of the stereotactic frame.

One important restriction applies to Equation 15. The four $\left(u, v, w\right)$ coordinates $\left(u_{1},v_{1},w_{1}\right)$, $\left(u_{2},v_{2},w_{2}\right)$, $\left(u_{3},v_{3},w_{3}\right)$, and $\left(u_{4},v_{4},w_{4}\right)$ must not be coplanar; hence, these four $\left(u, v, w\right)$ coordinates must not be obtained from a single plane, such as an axial plane that comprises four fiducials. Thus, for example, an acceptable set of $\left(u, v, w\right)$ coordinates would comprise three $\left(u, v, w\right)$ coordinates from three N-localizers that intersect an axial plane and one $\left(u, v, w\right)$ coordinate from the superior N-localizer that intersects a coronal plane. This restriction is similar to the restriction that applies to Equation 3, *i.e.*, three non-collinear $\left(u, v\right)$ coordinates must be used in Equation 3 [7].

In Equation 15, the matrix elements $x_1$ through $z_4$ as well as the matrix elements $u_1$ through $w_4$ are known. The matrix elements $m_{11}$ through $m_{43}$ are unknown; hence, it is possible (and tempting) to invert Equation 15 in order to solve for these unknown elements of the transformation matrix in a manner similar to Equation 4. However, a more useful solution may be obtained by applying the method of least squares [14] to more than four sets of fiducials because of the robustness of that method and the usefulness of the correlation coefficients that it provides.

For voxel data, a useful set of fiducials would comprise ten fiducials: four fiducials from an axial plane, three fiducials from a sagittal plane and three fiducials from a coronal plane, where the target point $P_T$ is visualized in all three planes. The following equation transforms ten $\left(u,v,w\right)$ coordinates from the three-dimensional coordinate system of the voxel data to create ten $\left(x,y,z\right)$ coordinates in the three-dimensional coordinate system of the stereotactic frame

$$\begin{bmatrix} x_i & y_i & z_i & 1 \end{bmatrix} = \begin{bmatrix} u_i & v_i & w_i & 1 \end{bmatrix} \begin{bmatrix} m_{11} & m_{12} & m_{13} & 0 \\ m_{21} & m_{22} & m_{23} & 0 \\ m_{31} & m_{32} & m_{33} & 0 \\ m_{41} & m_{42} & m_{43} & 1 \end{bmatrix}\;\;\;\;\;\;\;\;\;\;\left(16\right)$$

In Equation 16, the subscript $i$ selects one of the ten fiducials. The equations that are required for least-squares minimization are obtained by first expanding the matrix multiplication of Equation 16 and expressing the result for the matrix elements $x_i$, $y_i$, and $z_i$

$$\begin{matrix} x_i = u_i m_{11} + v_i m_{21} + w_i m_{31} + m_{41} \\ y_i = u_i m_{12} + v_i m_{22} + w_i m_{32} + m_{42} \\ z_i = u_i m_{13} + v_i m_{23} + w_i m_{33} + m_{43} \end{matrix}\;\;\;\;\;\;\;\;\;\;\left(17\right)$$

In the presence of error, Equation 17 may be rearranged to express the errors in $x_i$, $y_i$, and $z_i$ as $\delta x_i$, $\delta y_i$, and $\delta z_i$, respectively

$$\begin{matrix} \delta x_i = x_i - u_i m_{11} - v_i m_{21} - w_i m_{31} - m_{41} \\ \delta y_i = y_i - u_i m_{12} - v_i m_{22} - w_i m_{32} - m_{42} \\ \delta z_i = z_i - u_i m_{13} - v_i m_{23} - w_i m_{33} - m_{43} \end{matrix}\;\;\;\;\;\;\;\;\;\;\left(18\right)$$

In order to minimize these errors via the method of least squares, the equations for $\delta x_i$, $\delta y_i$ and $\delta z_i$ are squared to obtain the error functions $E_x$, $E_y$, and $E_z$

$$\begin{matrix} E_x \left(m_{11}, m_{21}, m_{31}, m_{41}\right) = \sum{ \left(x_i - u_i m_{11} - v_i m_{21} - w_i m_{31} - m_{41} \right)^2} \\ E_y \left(m_{12}, m_{22}, m_{32}, m_{42} \right) = \sum{ \left(y_i - u_i m_{12} - v_i m_{22} - w_i m_{32} - m_{42} \right)^2} \\ E_z \left(m_{13}, m_{23}, m_{33}, m_{43} \right) = \sum{ \left(z_i - u_i m_{13} - v_i m_{23} - w_i m_{33} - m_{43} \right)^2} \end{matrix}\;\;\;\;\;\;\;\;\;\;\left(19\right)$$

The following discussion illustrates minimization of the error function $E_x$; minimization of the error functions $E_y$ and $E_z$ is performed in an analogous manner. At the minimum of a function, all of the derivatives are equal to zero. Evaluating the derivatives $\partial E_x / \partial m_{11}$, $\partial E_x / \partial m_{21}$, $\partial E_x / \partial m_{31}$, and $\partial E_x / \partial m_{41}$ and setting the resulting expressions for these derivatives to zero yields

$$\begin{matrix} \partial E_x / \partial m_{11} = \sum{ 2 \left(x_i - u_i m_{11} - v_i m_{21} - w_i m_{31} - m_{41} \right) u_i } = 0 \\ \partial E_x / \partial m_{21} = \sum{ 2 \left(x_i - u_i m_{11} - v_i m_{21} - w_i m_{31} - m_{41} \right) v_i } = 0 \\ \partial E_x / \partial m_{31} = \sum{ 2 \left(x_i - u_i m_{11} - v_i m_{21} - w_i m_{31} - m_{41} \right) w_i } = 0 \\ \partial E_x / \partial m_{41} = \sum{ 2 \left(x_i - u_i m_{11} - v_i m_{21} - w_i m_{31} - m_{41} \right) } = 0 \end{matrix}\;\;\;\;\;\;\;\;\;\;\left(20\right)$$

Simplifying and rearranging the above equations for the derivatives yields a system of four simultaneous linear equations of the four unknowns $m_{11}$, $m_{21}$, $m_{31}$, and $m_{41}$

$$\begin{matrix} m_{11} \sum{{u_i}^2} + m_{21} \sum {u_i v_i} + m_{31} \sum {u_i w_i} + m_{41} \sum{ u_i} = \sum{u_i x_i} \\ m_{11} \sum{u_i v_i} + m_{21} \sum{{v_i}^2} + m_{31} \sum {v_i w_i} + m_{41} \sum{ v_i} = \sum{v_i x_i} \\ m_{11} \sum{u_i w_i} + m_{21} \sum{v_i w_i} + m_{31} \sum{{w_i}^2} + m_{41} \sum{w_i} = \sum{w_i x_i} \\ m_{11} \sum{u_i} + m_{21} \sum{v_i} + m_{31} \sum{w_i} + m_{41} n = \sum{x_i} \end{matrix}\;\;\;\;\;\;\;\;\;\;\left(21\right)$$

where $n$ is the number of sets of fiducials; in this case, $n = 10$. These simultaneous linear equations may be solved using Cramer's rule [15] or preferably Gauss elimination [16] to yield the matrix elements $m_{11}$, $m_{21}$, $m_{31}$, and $m_{41}$ that minimize the error function $E_x$. The error functions $E_y$ and $E_z$ are minimized in a similar manner to obtain the matrix elements $m_{12}$, $m_{22}$, $m_{32}$, and $m_{42}$ and the matrix elements $m_{13}$, $m_{23}$, $m_{33}$, and $m_{43}$, respectively.

Once the elements of the transformation matrix have been calculated as discussed above, the transformation matrix may be used as follows to transform the $\left(u_{T},v_{T},w_{T}\right)$ coordinates of the target point $P_{T}$ from the three-dimensional coordinate system of the voxel data into the three-dimensional coordinate system of the stereotactic frame to obtain the $\left(x_{T},y_{T},z_{T}\right)$ coordinates of the analogous target point ${P}'_{T}$

$$\begin{bmatrix} x_T & y_T & z_T & 1 \end{bmatrix} = \begin{bmatrix} u_T & v_T & w_T & 1 \end{bmatrix} \begin{bmatrix} m_{11} & m_{12} & m_{13} & 0 \\ m_{21} & m_{22} & m_{23} & 0 \\ m_{31} & m_{32} & m_{33} & 0 \\ m_{41} & m_{42} & m_{43} & 1 \end{bmatrix}\;\;\;\;\;\;\;\;\;\;\left(22\right)$$

The accuracy of the calculation of the transformation matrix elements, and hence the accuracy of the transformation of the $\left(u_{T},v_{T},w_{T}\right)$ coordinates, is indicated by three correlation coefficients $r_x$, $r_y$, and $r_z$ that express how well the right-hand sides of Equation 17 estimate the left-hand sides of that equation [26]. Taking the first of Equations 17 as an example, the correlation coefficient $r_x$ that measures the correlation between the right-hand side

$$\epsilon_i = m_{11}u_i + m_{21}v_i + m_{31}w_i + m_{41}\;\;\;\;\;\;\;\;\;\;\left(23\right)$$

and the left-hand side $x_i$ is calculated as [17]

$$r_x = \frac{n \sum {\epsilon_i x_i} - \sum {\epsilon_i} \sum {x_i}}{\sqrt{ n \sum {\epsilon_i}^2 - \left( \sum \epsilon_i\right )^2 } \sqrt{ n\sum {x_i}^2 - \left( \sum x_i\right )^2}}\;\;\;\;\;\;\;\;\;\;\left(24\right)$$

The correlation coefficients $r_y$ and $r_z$ are calculated in an analogous manner. The three correlation coefficients $r_x$, $r_y$, and $r_z$ may be used to calculate the correlation coefficient $r_{xyz}$, as shown in Equation 14.

The solution to Equation 21 provides a method for transforming $\left(u,v,w\right)$ coordinates from the three-dimensional coordinate system of voxel data, which are obtained via volume imaging, to the three-dimensional coordinate system of the stereotactic frame to produce $\left(x,y,z\right)$ coordinates. These equations require the use of four or more pairs of non-coplanar $\left(u,v,w\right)$ and $\left(x,y,z\right)$ coordinates. A useful set of $\left(u,v,w\right)$ and $\left(x,y,z\right)$ coordinates may be obtained from axial, sagittal, and coronal planes, in which the target point $P_T$ is visualized, by selecting the $\left(u,v,w\right)$ coordinates then calculating the $\left(x,y,z\right)$ coordinates via Equation 1 or Equation 2. Note that although axial, sagittal and coronal planes in which the target point is visualized would appear to be the most useful of the image planes, there is no requirement to include these particular planes in the calculation of the transformation matrix via solution of Equation 21. Because the transformation matrix transforms $\left(u,v,w\right)$ coordinates from the three-dimensional coordinate system of the voxel data to produce $\left(x,y,z\right)$ coordinates in the three-dimensional coordinate system of the stereotactic frame, all of the $\left(u,v,w\right)$ coordinates from the voxel data are transformed into $\left(x,y,z\right)$ coordinates, independent of the planes from which the $\left(u,v,w\right)$ coordinates are selected for application of Equations 1, 2, and 21.

An additional application of the solution to Equation 21 is the calculation of the correlation coefficients $r_x$, $r_y$, $r_z$, and $r_{xyz}$ that provide a measure of the accuracy of the transformation. The accuracy of the transformation is degraded by nonlinear distortion to which MR data are susceptible.

In view of the susceptibility of MR to nonlinear distortion, an assessment of the nonlinear distortion may be improved using additional $\left(u,v,w\right)$ and $\left(x,y,z\right)$ coordinates that may be obtained from axial, sagittal and coronal planes that do not include the target point $P_T$. These additional planes could be chosen from throughout the volume image; their $\left(u,v,w\right)$ and $\left(x,y,z\right)$ coordinates would contribute to the calculation of the correlation coefficients $r_x$, $r_y$, $r_z$, and $r_{xyz}$ and thereby provide a measure of the presence of nonlinear distortion within the volume image.

MR scanners are equipped with small "shimming" electromagnets that are used to improve the homogeneity of the magnetic field and, hence, improve the linearity of MR images. Because the optimum shim settings differ between patients, the optimum shim settings should be determined for each patient individually. A MR localizer frame could assist in the determination of the optimum shim settings as follows. A volume image that comprises voxel data would be obtained for a patient wearing a MR localizer frame, then $\left(u,v,w\right)$ coordinates would be chosen from throughout the volume image. The corresponding $\left(x,y,z\right)$ coordinates would be calculated from the $\left(u,v,w\right)$ coordinates via Equation 2. The $\left(u,v,w\right)$ and $\left(x,y,z\right)$ coordinates would be used to construct Equation 21. The solution to Equation 21 would yield the matrix elements $m_{11}$ through $m_{43}$. These matrix elements and the $\left(u,v,w\right)$ and $\left(x,y,z\right)$ coordinates would be used to calculate the correlation coefficients $r_x$, $r_y$, $r_z$, and $r_{xyz}$ via Equations 23-24. These correlation coefficients would provide an analysis of the linearity of the voxel data and thus provide an indication of whether the quality of the shimming procedure was sufficient to permit MR-guided stereotactic surgery.

## Conclusions

This article presents the mathematics that permit the transformation of $\left(u,v\right)$ coordinates from the two-dimensional coordinate system of a tomographic image to the three-dimensional coordinate system of the stereotactic frame to produce $\left(x,y,z\right)$ coordinates. In addition, this article describes the mathematics that permit the transformation of $\left(u,v,w\right)$ coordinates from the three-dimensional coordinate system of volume image data, which are obtained via MR imaging, to the three-dimensional coordinate system of the stereotactic frame to produce $\left(x,y,z\right)$ coordinates.

When applied to four or more N-localizers, these mathematics permit the calculation of the correlation coefficients $r_x$, $r_y$, $r_z$, and $r_{xyz}$ that provide a statistical measure of the presence of nonlinear distortion in the image data. Because image data that are obtained via MR imaging are susceptible to nonlinear distortion, these correlation coefficients may be used to indicate whether a particular MR image is sufficiently free of nonlinear distortion to qualify for MR-guided stereotactic surgery.

## Figures and Tables

**Figure 10:**
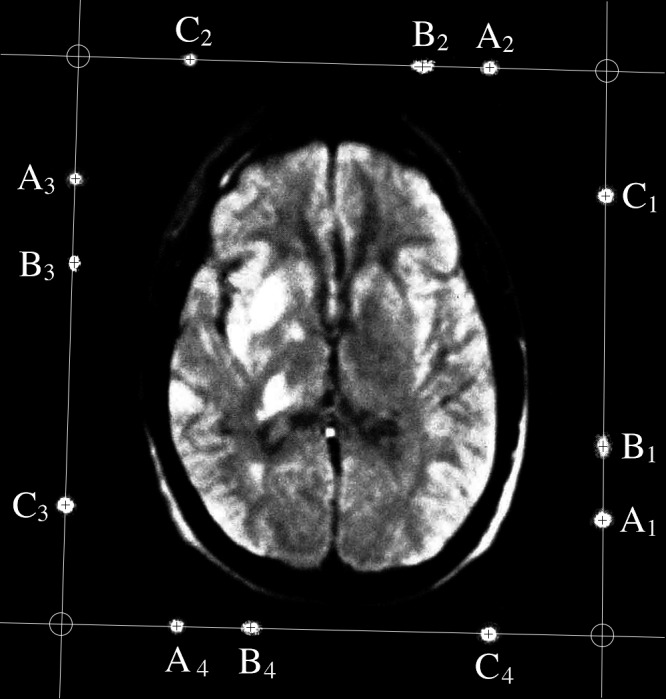
MR Image with Four Sets of Fiducials MR image of a patient to whom a first-generation BRW MR localizer frame (Radionics Inc., Burlington, MA) is attached. Four N-localizers create four sets of three collinear fiducials $\left\{ \mathrm{A_1,B_1,C_1} \right\}$, $\left\{ \mathrm{A_2,B_2,C_2} \right\}$, $\left\{ \mathrm{A_3,B_3,C_3} \right\}$, and $\left\{ \mathrm{A_4,B_4,C_4} \right\}$. The black cross hairs indicate the centers of the fiducials. For each set of three collinear fiducials, the centers of the fiducials define a line. The four lines intersect at the positions designated by open circles that indicate where the cross sections of vertical rods would create fiducials if vertical rods existed at the vertical edges of the cube. Adapted from [11].

**Figure 11:**
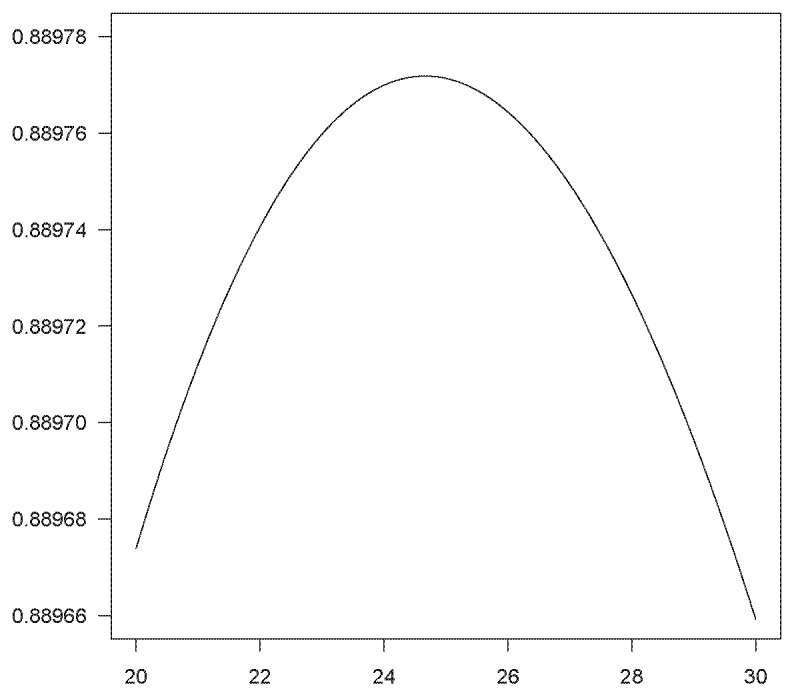
Plot of $r_{xyz}$ vs. the Separation (cm) Between Rods $\mathrm A$ and $\mathrm C$ The plot of $r_{xyz}$ vs. the separation between rods $\mathrm A$ and $\mathrm C$ reveals a maximum in $r_{xyz}$ near a separation of 25 cm. The range of $r_{xyz}$ is limited to $0.88966 \le r_{xyz} \le 0.88977$; hence, $r_{xyz}$ is only weakly dependent on the separation between rods $\mathrm A$ and $\mathrm C$.

## Appendices

### Appendix 1: Mathematical Model of the Stereotactic Frame in [9]

In order to calculate a transformation matrix from the data in Table 1, the $\left(x,y,z\right)$ coordinates of the points at both ends of each of the four diagonal rods $\mathrm B_1$, $\mathrm B_2$, $\mathrm B_3$, and $\mathrm B_4$ must be known in the three-dimensional coordinate system of the stereotactic frame that is visible in Figure 7 [9]. These $\left(x,y,z\right)$ coordinates are required to calculate the point of intersection between the long axis of each diagonal rod $\mathrm B_\mathrm i$ and the tomographic section via Equation 2. The author does not have access to that stereotactic frame, so the $\left(x,y,z\right)$ coordinates of the points at both ends of the four diagonal rods are not known.

However, it is possible to assign the $\left(x,y,z\right)$ coordinates of these eight points as follows. The stereotactic frame is designed such that the four N-localizers lie on the faces of a cube and the four diagonal rods $\mathrm B_1$, $\mathrm B_2$, $\mathrm B_3$, and $\mathrm B_4$ completely subtend the faces of that cube [9]. The vertical edges of the cube are vertical rods whose cross sections create the fiducials $\mathrm A_1$, $\mathrm A_2$, $\mathrm A_3$, and $\mathrm A_4$ in Figure 7. Hence, the length of an edge of the cube corresponds to the distance between two adjacent fiducials $\mathrm A$, for example, between $\mathrm A_1$ and $\mathrm A_2$. Thus, a sufficient mathematical model of the stereotactic frame is a cube whose edges are arbitrarily chosen to be 30 cm long and whose vertical rods $\mathrm A_1 = \mathrm C_4$, $\mathrm A_2 = \mathrm C_1$, $\mathrm A_3 = \mathrm C_2$ and $\mathrm A_4 = \mathrm C_3$ bracket the diagonal rods $\mathrm B_1$, $\mathrm B_2$, $\mathrm B_3$, and $\mathrm B_4$, respectively, as shown in Figure 2a. Consequently, the $\left(x,y,z\right)$ coordinates of the points at the ends of each diagonal rod $\mathrm B_\mathrm i$ may be assigned from the points at the ends of the bracketing vertical rods $\mathrm A_\mathrm i$ and $\mathrm C_\mathrm i$. For example, the beginning point of diagonal rod $\mathrm B_1$ is assigned from the point at the top of vertical rod $\mathrm A_1$, and the end point of diagonal rod $\mathrm B_1$ is assigned from the point at the bottom of vertical rod $\mathrm C_1$. The beginning and end points of the diagonal rods $\mathrm B_2$, $\mathrm B_3$, and $\mathrm B_4$ are assigned in a similar manner.

### Appendix 2: Mathematical Model of the Stereotactic Frame in [11]

In order to calculate a transformation matrix from the data in Table 3, the $\left(x,y,z\right)$ coordinates of the points at both ends of each of the four diagonal rods $\mathrm B_1$, $\mathrm B_2$, $\mathrm B_3$, and $\mathrm B_4$ must be known in the three-dimensional coordinate system of the stereotactic frame that is visible in Figure 8 [11]. These $\left(x,y,z\right)$ coordinates are required to calculate the point of intersection between the long axis of each diagonal rod $\mathrm B_\mathrm i$ and the tomographic section via Equation 2. The author does not have access to that stereotactic frame, so the $\left(x,y,z\right)$ coordinates of the points at both ends of the four diagonal rods are not known.

Moreover, the geometry of the stereotactic frame presents some challenges to assigning the $\left(x,y,z\right)$ coordinates of these eight points. The stereotactic frame is designed such that the four N-localizers lie on the faces of a cube. However, although the four diagonal rods $\mathrm B_1$, $\mathrm B_2$, $\mathrm B_3$, and $\mathrm B_4$ completely subtend the faces of that cube in the direction perpendicular to the central plane of the tomographic section, the diagonal rods only partially subtend the faces of the cube in the direction within the central plane of the tomographic section [11]. Also, the vertical edges of the cube do not coincide with vertical rods and hence do not create fiducials in Figure 8. Thus, the length of an edge of the cube does not correspond to the distance between two such fiducials.

In an attempt to develop a sufficient mathematical model of the stereotactic frame, the positions of the vertical edges of the cube are estimated from Figure 10, which depicts the same MR image as Figure 8. In Figure 10, each of the four sets of fiducials $\left\{ \mathrm{A_1,B_1,C_1} \right\}$, $\left\{ \mathrm{A_2,B_2,C_2} \right\}$, $\left\{ \mathrm{A_3,B_3,C_3} \right\}$, and $\left\{ \mathrm{A_4,B_4,C_4} \right\}$ comprises three collinear fiducials. Each set of three collinear fiducials lies on a different edge of a square. From each set of collinear fiducials, the points at the centers of the three fiducials define a line that describes a different edge of the square. These four lines intersect at the positions designated by the open circles in Figure 10 that indicate where the cross sections of vertical rods would create fiducials if vertical rods existed at the vertical edges of a cube.

From the positions of the open circles in Figure 10, it is possible to assign the $\left(x,y,z\right)$ coordinates of the points at the ends of each diagonal rod $\mathrm B_\mathrm i$. In this figure, the cross sections of the faces of the cube correspond to the line segments between the open circles. These line segments are arbitrarily chosen to be 30 cm long. From this length, the separation between each rod $\mathrm A_\mathrm i$ and the corresponding rod $\mathrm C_\mathrm i$ is estimated to be 23 cm.

Because each pair of vertical rods $\mathrm A_\mathrm i$ and $\mathrm C_\mathrm i$ bracket a diagonal rod $\mathrm B_\mathrm i$, as shown in Figure 2a, the $\left(x,y,z\right)$ coordinates of the points at the ends of each diagonal rod $\mathrm B_\mathrm i$ may be assigned from the points at the ends of the bracketing vertical rods $\mathrm A_\mathrm i$ and $\mathrm C_\mathrm i$. For example, the beginning point of diagonal rod $\mathrm B_1$ is assigned from the point at the top of vertical rod $\mathrm A_1$, and the end point of diagonal rod $\mathrm B_1$ is assigned from the point at the bottom of vertical rod $\mathrm C_1$. The beginning and end points of the diagonal rods $\mathrm B_2$, $\mathrm B_3$, and $\mathrm B_4$ are assigned in a similar manner.

It is possible that estimation of the positions of the vertical rods $\mathrm A_\mathrm i$ and $\mathrm C_\mathrm i$, as discussed above, could influence the accuracy of transformation of the $\left(u_T,v_T\right)$ coordinates of the target point $P_T$ into the $\left(x_T,y_T,z_T\right)$ coordinates of the analogous target point ${P}'_T$. The correlation coefficient $r_{xyz}$, which measures this accuracy, is calculated from the points at the ends of the diagonal rods $\mathrm B$ via Equations 2, 13, and 14. The points at the ends of each diagonal rod $\mathrm B_\mathrm i$ are assigned from the points at the ends of the bracketing vertical rods $\mathrm A_\mathrm i$ and $\mathrm C_\mathrm i$. Hence, the correlation coefficient $r_{xyz}$ may be a function of the separation between rods $\mathrm A$ and $\mathrm C$. Moreover, the robustness of $r_{xyz}$ may be influenced by the fact that the separation between rods $\mathrm A$ and $\mathrm C$ is estimated from Figure 10. To elucidate these potential dependencies, a plot of $r_{xyz}$ versus the separation between rods $\mathrm A$ and $\mathrm C$ is shown in Figure 11. This plot reveals a maximum in $r_{xyz}$ near a separation of 25 cm. In addition, this plot demonstrates that $r_{xyz}$ is only weakly dependent on the separation between rods $\mathrm A$ and $\mathrm C$, as indicated by the limited range of $r_{xyz}$ from 0.88966 to 0.88977. Hence, the robustness of $r_{xyz}$ is not influenced by the separation between rods $\mathrm A$ and $\mathrm C$.

An iterative search for the optimum separation, which corresponds to the maximum in $r_{xyz}$, found an optimum separation of 24.67 cm. This optimum separation was used to calculate the $\left(x_T,y_T,z_T\right)$ coordinates for the target points that are shown in Table 4.
